# Addressing environmental misperceptions for nature recovery

**DOI:** 10.1111/cobi.70157

**Published:** 2025-10-18

**Authors:** Shuo Gao, Sophus O. S. E. zu Ermgassen, Joseph W. Bull, E. J. Milner‐Gulland

**Affiliations:** ^1^ Interdisciplinary Centre for Conservation Science, Department of Biology University of Oxford Oxford UK; ^2^ School of Public Affairs University of Science and Technology of China Hefei China

**Keywords:** amnesia, change blindness, citizen science, cognitive biases, conservation psychology, environmental education, human–nature relationships, memory illusion, amnesia, ceguera al cambio, ciencia ciudadana, educación ambiental, ilusión de la memoria, psicología de la conservación, relaciones entre el ser humano y la naturaleza, sesgos cognitivos

## Abstract

A poorly understood and systemic challenge to global conservation agreements is shifting baseline syndrome (SBS), wherein people misperceive the extent to which nature has changed. This can diminish societal expectations for nature recovery. We broadened the conceptual framing of SBS beyond the more common elements of nature loss to include nature recovery and the cognitive mechanisms underlying misperceptions. To demonstrate the utility of the framework, we surveyed people living in Qunli New Town, Harbin, China. We first conducted in‐depth interviews with a semirandomized sample of 42 people to qualitatively explore the diverse manifestations of environmental misperceptions and the cognitive processes that drive misperceptions in the study area. We then administered an online survey of 1018 people to quantitatively estimate the scale of SBS and identify factors affecting individual misperceptions. The accuracy of these perceptions was determined by comparing participants’ reported environmental conditions with actual measurements. Inaccurate perceptions were linked to media‐based (e.g., television) environmental information sources; direct interaction with nature did not foster ecological understanding in most cases; depth of personal engagement (e.g., interest in local nature and time spent per visit) was associated with such understanding; and cognitive errors underlying environmental misperceptions, including errors of omission and commission, were related to cognitive processes, such as sensation, attention, learning, thinking, and memory. More nuanced, place‐based strategies are needed that explicitly address the structural and cognitive dimensions of environmental misperceptions. Minimizing such misperceptions is important so that people affected by environmental change can better respond to it. This is essential for pursuing resilient, sustainable, and inclusive societies under the Sustainable Development Goals and the Global Biodiversity Framework.

## Abstract (es)

Abordar las percepciones erróneas ambientales para la recuperación de la naturaleza

**Resumen:**

Un desafío sistémico y poco comprendido para los acuerdos de conservación mundiales es el síndrome de desplazamiento de la línea de base (SBS), en el que las personas perciben erróneamente el alcance del cambio que ha sufrido la naturaleza. Esto puede disminuir las expectativas de la sociedad respecto a la recuperación de la naturaleza. Ampliamos el marco conceptual del SBS más allá de los elementos más comunes de la pérdida de naturaleza para incluir la recuperación de la naturaleza y los mecanismos cognitivos subyacentes en las percepciones erróneas. Para demostrar la utilidad del marco, realizamos una encuesta a personas que viven en Qunli New Town, Harbin, China. En primer lugar, llevamos a cabo entrevistas en profundidad con una muestra semialeatoria de 42 personas para explorar cualitativamente las diversas manifestaciones de las percepciones erróneas sobre el medio ambiente y los procesos cognitivos que impulsan las percepciones erróneas en el área de estudio. Posteriormente, realizamos una encuesta en línea a 1018 personas para estimar cuantitativamente la magnitud del SBS e identificar los factores que influyen en las percepciones erróneas individuales. La precisión de estas percepciones se determinó comparando las condiciones ambientales comunicadas por los participantes con las mediciones reales. Las percepciones imprecisas se relacionaron con fuentes de información medioambiental basadas en los medios de comunicación (por ejemplo, la televisión); la interacción directa con la naturaleza no fomentó la comprensión ecológica en la mayoría de los casos; la profundidad del compromiso personal (por ejemplo, el interés por la naturaleza local y el tiempo dedicado a cada visita) se asoció con dicha comprensión; y los errores cognitivos subyacentes a las percepciones erróneas sobre el medio ambiente, incluidos los errores de omisión y comisión, se relacionaron con procesos cognitivos, como la sensación, la atención, el aprendizaje, el pensamiento y la memoria. Se requieren estrategias más matizadas y basadas en el sitio que aborden explícitamente las dimensiones estructurales y cognitivas de las percepciones erróneas sobre el medio ambiente. Es importante minimizar esas percepciones erróneas para que las personas afectadas por el cambio medioambiental puedan responder mejor a él. Esto es esencial para lograr sociedades resilientes, sostenibles e inclusivas en el marco de los Objetivos de Desarrollo Sostenible y el Marco Mundial para la Diversidad Biológica.

## INTRODUCTION

Nature recovery is a salient topic embraced globally by governments, businesses, financial institutions, and the conservation sector (Bull et al., 2020; Maron et al., 2024; zu Ermgassen et al., 2022). As nations commit to taking urgent action to support a nature‐positive future (Maron et al., 2024; zu Ermgassen et al., 2022), it is vital for the general public to accurately perceive trends in the natural environment so that they can hold governments and companies implementing nature recovery accountable. The sociopsychological phenomenon shifting baseline syndrome (SBS), wherein people misperceive the extent to which nature has been degraded or improved, can diminish societal expectations about how much nature recovery a society wants (Jarić et al., 2022; Manning et al., 2006; McClenachan et al., 2018; Miller, 2005; Ostergren et al., 2008; Papworth et al., 2009; Pauly, 1995; Soga & Gaston, 2018). Thus, it stands in the way of the major changes needed to establish a sustainable future for Earth (Díaz et al., 2019; Raworth, 2017; Richardson et al., 2023) and needs to be addressed as part of the path to nature recovery.

SBS can be experienced by a person in their own life or across generations; therefore, it is conceptualized as related to someone's age (Papworth et al., 2009). The literature largely focuses on research reporting resource users’ perceptions regarding changes in natural resources. However, SBS can occur across diverse environmental conditions, ecosystems, and stakeholders (Muldrow et al., 2020; Soga & Gaston, 2018), and its features may differ among societies (particularly if rapid social change accompanies ecological change). Although SBS provides a logical explanation for inaccurate perceptions of past environmental states, other mechanisms causing mismatches between perceptions and reality have been identified, for example, change blindness and memory illusion (Daw, 2010) (definitions in “METHODS”). Most previous studies of SBS relate to environmental degradation. Yet, it is also essential to determine how SBS manifests as nature recovers, because in some places, the environment is being restored for new generations (Lovell et al., 2020; Papworth et al., 2009; Passoni et al., 2023; Roman et al., 2015).

Understanding and addressing SBS is ecologically and socially important (Ford et al., 2020; Hidalgo Pizango et al., 2022; Manning et al., 2006; Soga & Gaston, 2018, 2023; Soga et al., 2023; Swanson et al., 2021). Lack of reliable baseline information is a fundamental barrier to resolving a broad swathe of contemporary environmental problems (Papworth et al., 2009). Baselines established through perceptions that deviate from actual situations and baseline shifts can weaken the validity of participatory monitoring, community‐based conservation, and environmental education (Papworth et al., 2009; Swanson et al., 2021). Shifting baselines can normalize degraded conditions and create stereotypes of habitat needs that misinform conservation, leading to suboptimal outcomes, such as conserving species in areas with poor‐quality habitat or historically no habitat (Kerley et al., 2012, 2020; Kuemmerle et al., 2012). Such shifts limit opportunities for habitat restoration and can skew threat assessments and conservation priorities (Smith et al., 2024).

People's environmental perceptions shape their experiences of and responses to environmental degradation (Downing et al., 2019; Hackmann et al., 2014). Especially, SBS affects what individuals perceive as degraded environmental conditions, thus potentially dampening proenvironmental attitudes and behaviors (e.g., putting pressure on government or businesses to undertake restoration) (Jones et al., 2020; Soga & Gaston, 2016, 2018). A lack of awareness of environmental improvements could also reduce the strength of calls for further restoration (Hausmann et al., 2016; Shoyama et al., 2013).

When SBS results in perceptual inertia (Soga & Gaston, 2018), it can extend to institutions and foster a “preemptive constraint of vision” (Manning et al., 2006). Anticipating limited public or political support for bold interventions, decision makers may favor incremental or socially acceptable measures. Although such approaches may seem pragmatic, they risk impeding effective responses to environmental decline, thereby perpetuating environmental degradation, missing opportunities, and misdirecting resources needed for transformative change.

The social consequences of environmental misperceptions can be far‐reaching by impacting critical ecosystem services, such as access to clean water and basic materials, protection from natural hazards, and provision of recreation and cultural benefits (Bryce et al., 2016; Díaz et al., 2006; MEA, 2005). Such effects are not only local or immediate but also intertemporal, emerging across generations, and telecoupled, driven by distant socioecological interactions (Agyeman, 2013; Robles‐Zavala & Reynoso, 2018). Thus, those most affected by environmental losses are not necessarily those who possess inaccurate or inadequate environmental knowledge.

For these reasons, researchers have called for better understanding of how people perceive and recall environmental change (Daw, 2010; Essl et al., 2015; Jarić et al., 2022) and of how to prevent and reverse SBS (Soga & Gaston, 2018). Policy solutions for addressing the consequences of SBS have been proposed. For instance, Manning et al. (2006) suggest ambitious goals and backcasting to counter the “ratcheting down” of societal expectations for nature recovery. To our knowledge, few studies have explicitly treated SBS as a sociopsychological phenomenon. Crucially, interventions to address environmental misperceptions, like those in other psychological domains, can be difficult to design effectively and scale up without a good understanding of the psychological mechanisms behind misperceptions. As far as we are aware, there is little to no research that explicitly addresses the psychological mechanisms behind mismatches between perceived and actual environmental change. To extend SBS beyond the natural resource context and to consider how SBS interacts with social change (Soga & Gaston, 2018), we aimed to devise a framework that can be applied to the study of environmental misperceptions in any local context.

## METHODS

### Framework for studying environmental misperceptions

**FIGURE 1:**
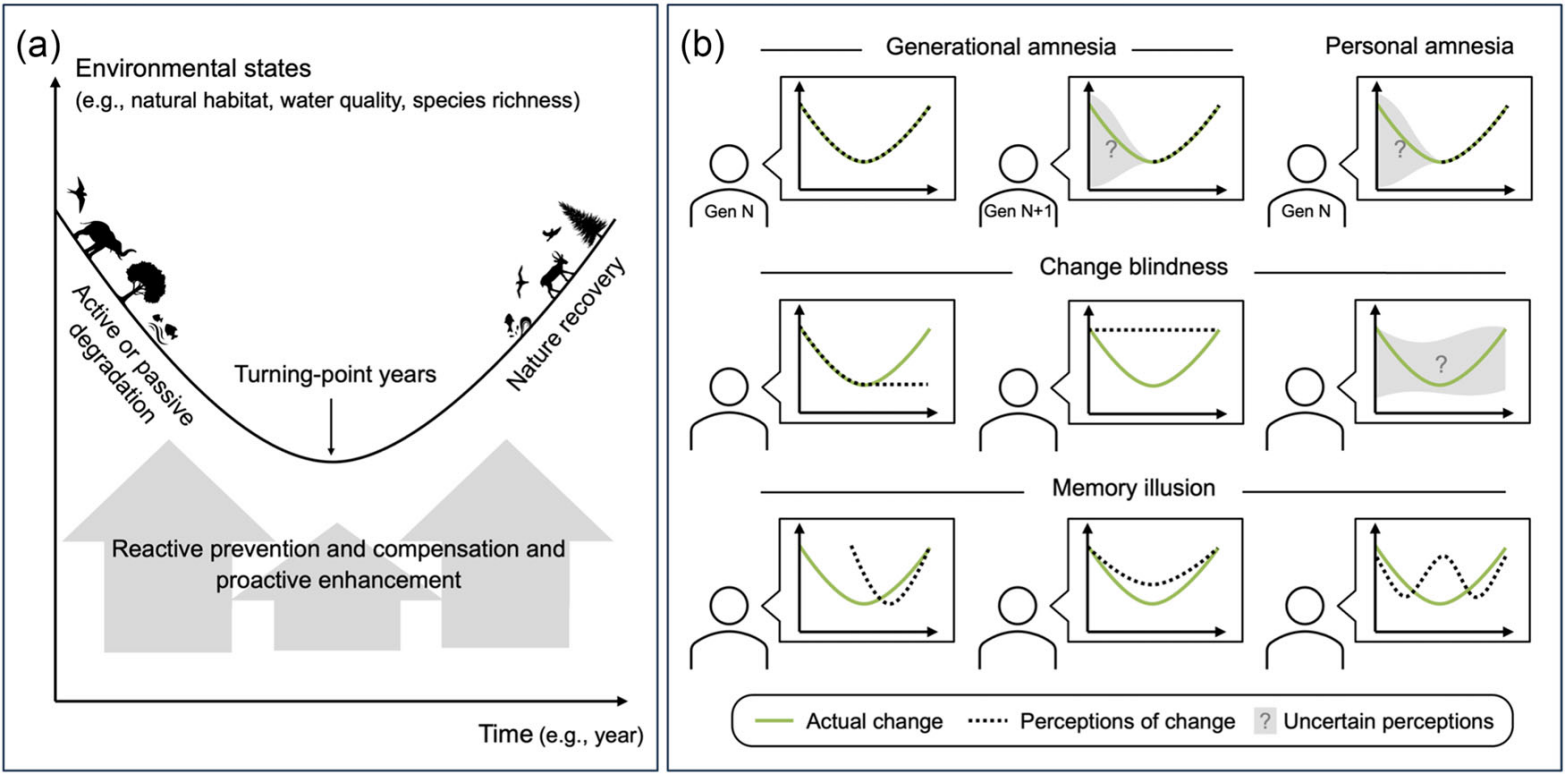
A conceptual model of perceptions and misperceptions of environmental baselines in a social–ecological system: (a) environmental trend in a local ecological system and the general trends in area of greenspace, water quality, and fish species richness in Qunli New Town and Songhua River (Harbin section) and (b) local people's multiple perceptions and misperceptions of change in the local social–ecological system relative to the typology of the 4 mechanisms for environmental misperception (i.e., shifting baseline syndrome related to generational amnesia or personal amnesia, change blindness, memory illusion).

We sought to conceptualize people's environmental misperceptions by drawing on and extending the typology of Papworth et al. (2009) (Figure 1). SBS encompasses generational and personal amnesia (Jones et al., 2020; Papworth et al., 2009) (Figure 1b). Generational amnesia refers to the phenomenon in which individuals shape their perceptions based solely on their own experiences and do not transmit these experiences to succeeding generations (Papworth et al., 2009; Pauly, 1995; Straka et al., 2025). Personal amnesia characterizes individuals adjusting their perception of what is normal to the extent that even those who encountered different circumstances in the past come to believe that present conditions mirror those of the past (Jones et al., 2020; Papworth et al., 2009). By definition, SBS also encompasses change blindness and memory illusion. With change blindness, individuals exhibit an inability to discern a change, partially or entirely (Jensen et al., 2011; Papworth et al., 2009; Simons & Levin, 1997). A memory illusion, a false recollection of past events, can distort an individual's perception of the timing, magnitude, and nature of change (Jacoby & Whitehouse, 1989; Papworth et al., 2009; Roediger III, 1996).

Studies exploring environmental misperceptions in a social–ecological system need to compare people's environmental perceptions with actual environmental data (Papworth et al., 2009). At the societal level, it is important to determine, for example, who experiences environmental misperception, what factors (e.g., direct or indirect experience with nature, sociodemographic and economic characteristics) explain the degree of environmental misperception, and how different types of misperceptions take place in a specific context (Figure 1b). This knowledge could provide insights that inform targeted interventions to prevent future or address past misperception.

**FIGURE 2:**
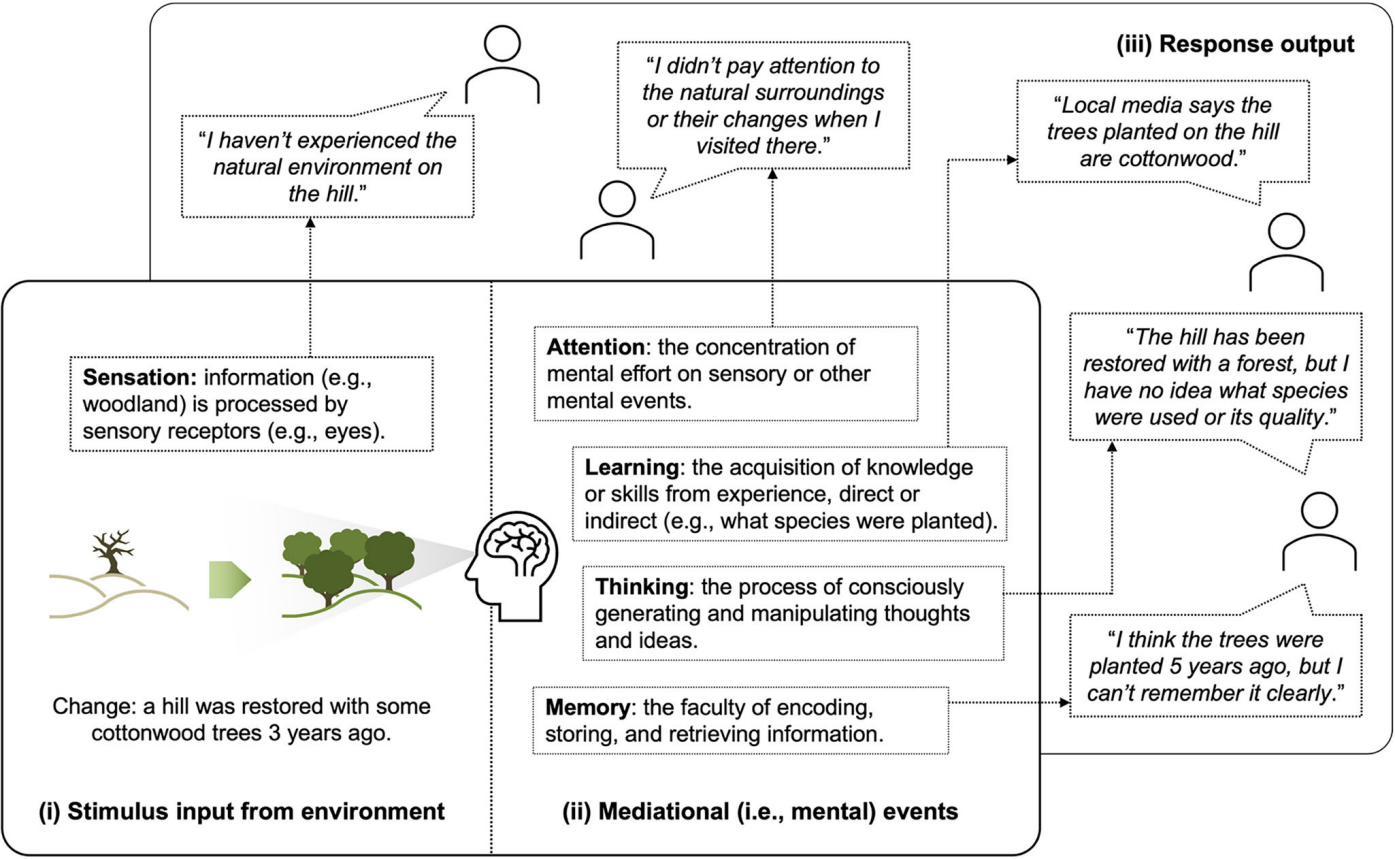
A qualitative conceptual model of the general cognitive processes that can lead to shifting baseline syndrome shown in Figure 1. The diagram does not include all mediational or mental events or all possible responses from the local stakeholders. Processes can also entail other actions (e.g., eyeball tracking) aside from those illustrated. Responses in (iii) are hypothetical and provide conceptual structure.

From the perspective of cognitive psychology, knowing is a process, not just a product (Bruner, 1974). To take our enquiries further into psychological understanding of how cognitive events drive misperceptions, we applied information processing theory (Lindsay & Norman, 1972; Newell & Simon, 1972; Oppenheimer & Kelso, 2015; Reitman, 1964; Simon, 1978). This theory views the human mind as a processor of information, likening it to a computer. This widely accepted metaphor suggests the human mind receives, processes, stores, and retrieves information. We adopted this theory to our study of environmental misperceptions (Figure 2). According to the theory, environmental stimuli—such as changes in vegetation, water quality, species populations, and landscape alterations—serve as sensory inputs. In a bottom‐up process, the detected information is relayed to higher brain areas for further processing and interpretation. For example, when observing a flower in a particular habitat, bottom‐up processing involves the sensory system detecting features, such as shape, color, and smell, that are then integrated to recognize the flower.

Information processing can also be top‐down (Engel et al., 2001; Gilbert & Sigman, 2007; Makino & Komiyama, 2015; Teufel & Nanay, 2017; von Stein et al., 2000). Top‐down processing involves the influence of higher level cognitive processes, such as prior knowledge and expectations, on the processing and interpretation of sensory information. This means that cognitive processes can shape perception by, for example, guiding attention, biasing ideas, and filling in missing information. For example, if an individual expects to see a certain flower species (e.g., lavender) about which they have sufficient prior knowledge, their expectations may influence how they pay attention to other plant species in the area and can affect how they interpret ambiguous sensory information, leading them to perceive the flower even if it is partially obscured (Himberger et al., 2018; Yuille & Kersten, 2006). In contrast, if the individual has insufficient knowledge, top‐down processing can lead to biased perceptions, such as mistaking sage for lavender, due to the similarities between the 2 species.

We suggest that environmental misperceptions can be researched through the lens of information processing theory, given its well‐developed structure and explanatory power in accounting for the cognitive mechanisms behind environmental misperception. We recognize that alternative theoretical approaches—such as constructivist ones—can also offer valuable insights. For instance, in determining how attention is directed and shaped by context, cultural norms, and social learning, constructivist theories may be more appropriate for addressing questions about how individuals come to notice—or fail to notice—environmental changes due to sociocultural influences. We therefore encourage their use in complementary research, where appropriate. By clearly defining the scope and purpose of our framework, we aimed to provide a coherent cognitive account of environmental misperception while remaining open to interdisciplinary engagement.

Understanding people's environmental misperceptions at the societal level can inform the design of interventions to change these perceptions if the goal is to make perceptions as accurate as possible. Investigating misperception at the cognitive level allows one to understand the phenomenon more fundamentally and thus design more powerful interventions to address it. When designing interventions, distinguishing between errors of omission and commission can be helpful (Schacter, 1999, 2002, 2022). Errors of omission include the many cognitive processes that can lead to losses of information input or storage, for example, being inattentive or failing to remember situations through time. They may also block, for example, tip‐of‐the‐tongue memories (i.e., people know a piece of information, such as a past environmental feature, but struggle to recall it fully or accurately) (Brown, 1991; Maylor, 1990). Errors of commission mean people have inaccurate knowledge due to a range of biases that distort their recollections of the past. This might include being subject to misinformation that implants incorrect knowledge and being overconfident about one's ability to notice and understand environmental changes.

Sociodemographic and economic factors, such as background, income, cultural norms, and political beliefs, can greatly affect environmental misperceptions by influencing how individuals access, interpret, and retain information about environmental changes. Socioeconomic status, for instance, can affect exposure to environmental education. For example, lower‐income groups may prioritize immediate economic needs over long‐term environmental problems, potentially leading to gaps in awareness or distorted perceptions (Philippssen et al., 2017). Cultural norms can also play a role in framing environmental attitudes. Societies that emphasize harmony with nature may be more attuned to ecological shifts, whereas those valuing industrial progress might downplay environmental decline (Zeyer & Kelsey, 2013). Whether a study needs to control for these variables depends on the research targets. If the aim is to isolate universal mechanisms behind environmental misperceptions (e.g., inattention), then controlling for these variables helps distinguish between general psychological tendencies and context‐dependent influences. For example, if researchers want to test whether overconfidence in recalling past environmental conditions is a widespread phenomenon, they might statistically adjust for education or political ideology to rule out alternative explanations. Yet, if the goal is to describe how misperceptions vary across societies (e.g., whether urban and rural populations differ in their perceptions of bird biodiversity loss), then controlling for particular factors, such as income or political beliefs, might hide meaningful patterns. Instead, researchers should assess and report these differences to understand real‐world variation.

### Case study

**FIGURE 3:**
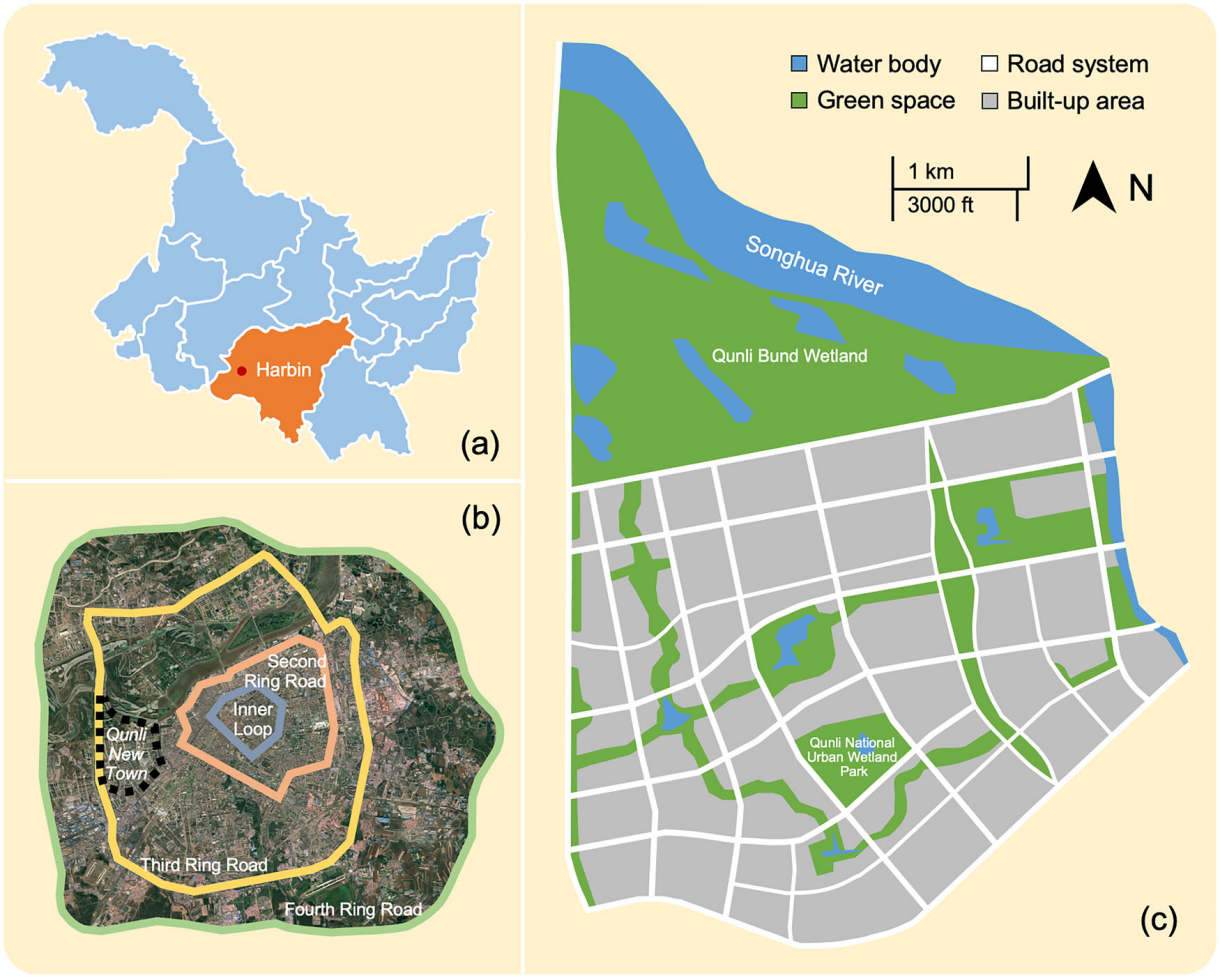
In Harbin City, Heilongjiang Province, China, the (a) location of Qunli New Town in Heilongjiang Province (45°44ʹ19.7ʺN 126°33ʹ01.9ʺE), (b) location of Qunli New Town in Harbin City, and (c) land‐use map of Qunli New Town in 2023, showing the newly created Qunli Bund Wetland and Qunli National Urban Wetland Park, both of which are replacing degraded wetlands, and green corridors connecting urban spaces. [Correction added on March 19, 2026 after first online publication: Figure 3 has been revised to correct the map scale.]

We applied our framework to a case study of Qunli New Town, Harbin, China, on the Songhua River (Harbin Section), a recently urbanized area, where multiple developments were implemented and were followed by projects to mitigate and compensate for development impacts (Figure 3). Substantial societal transformation marked the replacement of a rural population with an urban population. This novel setting allowed an in‐depth exploration of the interactions between societal and environmental changes in an increasingly typical scenario of development followed by ecological restoration and compensation (Bull & Strange, 2018; Damiens et al., 2021; Gao et al., 2023; Jacob et al., 2020; Li et al., 2021).

Harbin is the capital and the largest city of Heilongjiang Province and China's biggest provincial capital by land size. It is a key political, economic, and cultural hub in northeastern China. The population of Harbin quadrupled from 2.5 million in 1982 to 10 million in 2010. As a key part of the city's economic development scheme in the 2000s, the new town replaced the predominantly rural old town.

Numerous landscape‐level projects were implemented to improve existing natural areas and create new green spaces. Local ecological surveys suggested that the creation of the Qunli Bund Wetland led to its use by many species listed as threatened on the International Union for Conservation of Nature Red List, for example, the endangered Oriental stork (*Ciconia boyciana*) and the vulnerable white‐naped crane (*Grus vipio*). The Qunli National Urban Wetland Park (Figure 3c) was created by restoring a degraded marsh. Recognized by the UN Human Settlements Programme as an example of a prevention‐oriented measure (intervention to mitigate risks and prevent environmental harm before it occurs), the park safeguards native habitats, mitigates climate change impacts, and delivers cultural benefits for residents and tourists (Austin & Yu, 2016; UN‐Habitat, 2022; Zhu et al., 2020). Restoration of the river has increased populations of threatened fish species, for example, the critically endangered kaluga (*Huso dauricus*).

These measures resulted in a decline followed by an improvement in local environmental conditions. Local official documents (see Appendix S1) indicate a U‐shaped recovery in water quality (Appendix S2), with improvements beginning in 2009. They also demonstrate that fish species richness in the river rebounded following the improvement in water quality and that the number of fish species declined from 79 in the 1970s to 56 in 1990 and further to 34 in 2000. In 2020, after water quality improvements combined with fish restocking efforts the number of fish species rose back to 64 (Appendix S1). Although the available information is limited and relatively simple, it provides one way to reveal local environmental trends over time and represents the best data that could be obtained.

We employed a 2‐stage mixed‐methods approach (Yin, 2009). We used our exploratory study and official documentation to design questions asking respondents to report their knowledge of the environmental context and perceptions of environmental change, which could be compared against 3 ecological indicators for which we had real data on ecological change over time, including the area of natural habitat, water quality, and fish species richness. The questionnaire also asked respondents to identify specific plant species used in the implementation of compensation measures in the town, and their responses were compared with the species that were actually used.

In the first stage, we sought to qualitatively document local people's perceptions and misperceptions of environmental change in Qunli New Town and discern the cognitive processes involved. S.G. conducted semistructured interviews in the local language and dialect. Respondents were selected through semirandomized street‐by‐street sampling (main roads, including all parks) in new town (Ackrill et al., 2023; Igudia et al., 2022) and from the western and southern edges of the town, where residents of the old town were relocated.

S.G. lived in the new town from November 2022 to May 2023 and from August to September 2023, which allowed for informal conversations with local people, including the local authorities, and extensive field observations in local natural areas.

Potential participants were approached in public areas and asked to participate in the interviews. Our sampling therefore targeted residents found outdoors.

S.G. stopped interviewing people when a relative data saturation point was reached (Braun & Clarke, 2021; Gerson & Damaske, 2020; Glaser & Strauss, 1967; Small, 2009). Complete saturation is generally not possible because new information can always emerge from more interviews (Low, 2019; O'Reilly & Parker, 2013; Wray et al., 2007). Interviewing a new respondent nearly always brought up new perceptions of environmental features, for example a different bird behavior in a particular place. Thus, we aimed to achieve “depth and richness of analysis” (Small, 2009). This approach prioritized pragmatic saturation—balancing thoroughness with feasibility. Thus, interviews were halted once the responses provided evidence of whether SBS was an issue at the site and once new participants no longer provided new information relative to the cognitive mechanisms behind people's environmental misperceptions. We focused on respondents’ basic cognitive processes, including sensation, attention, learning, thinking, and memory (Kausler, 2012; Mesulam, 1998; Pisoni, 2000; Solso et al., 2005). In total, S.G. conducted 42 interviews, lasting from 25 min to 1.5 h. The respondent demographics reflected the age and gender composition in the latest (2020) census of the city.

We used thematic analyses to analyze the interview data, following the 6 phases of Braun and Clarke (2006). All content was transcribed and translated into English before coding. The translated content was retranslated from English back to Chinese with the help of local assistants to check accuracy and quality (Brislin, 1970, 1986). This process ensured maximal preservation of the original meaning and identified and corrected terminological discrepancies, small variations, and conceptual differences between language versions, for instance, preventing mistranslations, such as translating *environmental perception* as *environmental awareness*.

S.G. coded the SBS themes and cognitive components based on a deductive top‐down approach. The goal of the thematic analyses was to examine how cognitive processes can cause misperceptions of the natural environment. This exploration was general in nature, because the many subdivisions of a process and the complex interactions between processes, such as selective attention or sensory memory, were beyond the scope of the analysis.

In second stage, we used results from the interviews and official documents to design a questionnaire in which respondents were asked to report their knowledge of the environmental context and perceptions of environmental change. We compared respondents’ perceptions with 3 ecological indicators for which we had real data on change over time: amount of natural area, water quality, and fish species richness. The questionnaire also asked respondents to identify specific plant species used in the implementation of ecological compensation. Their responses were compared with the species that were actually used in the compensation.

Fourteen closed questions examined people's knowledge of the environment before and after land conversion. Two scores were generated. One that evaluated respondents’ knowledge of previous environmental states and one that evaluated knowledge of current environmental states. We counted the number of indicators for which their responses were aligned with the official data on ecological change over time. Respondents were also asked to state the date when they started to experience (live or move to) the study site (both the currently urban area and the river). In closed questions, respondents were asked to report their certainty that they had correctly answered the quiz questions and the reasons they felt uncertain (if applicable). We asked for reasons to uncover different cognitive drivers (Table 3).

The questionnaire also included components that assessed people's experience, including frequency of visits to the previous town, frequency of visits to the natural area in the new town, time spent per visit, level of interaction with nature, and knowledge sources (including direct, personal experience, and indirect means, including relatives and friends, printed media, TV, official websites or social media, and unofficial websites or social media). There was also a section of question on sociodemographic features.

The questionnaire survey was distributed through Wenjuanxing (http://www.wjx.cn/), one of China's largest online survey platforms that offers survey services (Rui, 2023; Wang et al., 2021; Zheng et al., 2024). In the first round, the online survey of people living in the study region was biased toward younger people. A second round targeted people living in the area over age 45. The final, combined sample size was 1326.

We examined the statistical differences between the results for the over 45s in the second round and the same age group in the earlier sample. We conducted 3 statistical tests based on the total scores the 2 groups obtained for correctly answering questions related to all environmental elements. The Wilcoxon rank‐sum test showed no significant difference in the medians (*W* = 22,960, *p* = 0.8618); Welch 2‐sample *t* test indicated no significant difference in the means (*t* = −0.13888, *p* = 0.8896); and Levene's test for homogeneity of variance revealed no significant difference in variances between the groups (*F* = 0.0332, *p* = 0.8556). Based on the results of these tests, there was no evidence of significant differences, suggesting that the 2 groups could be merged for further analyses.

**FIGURE 4:**
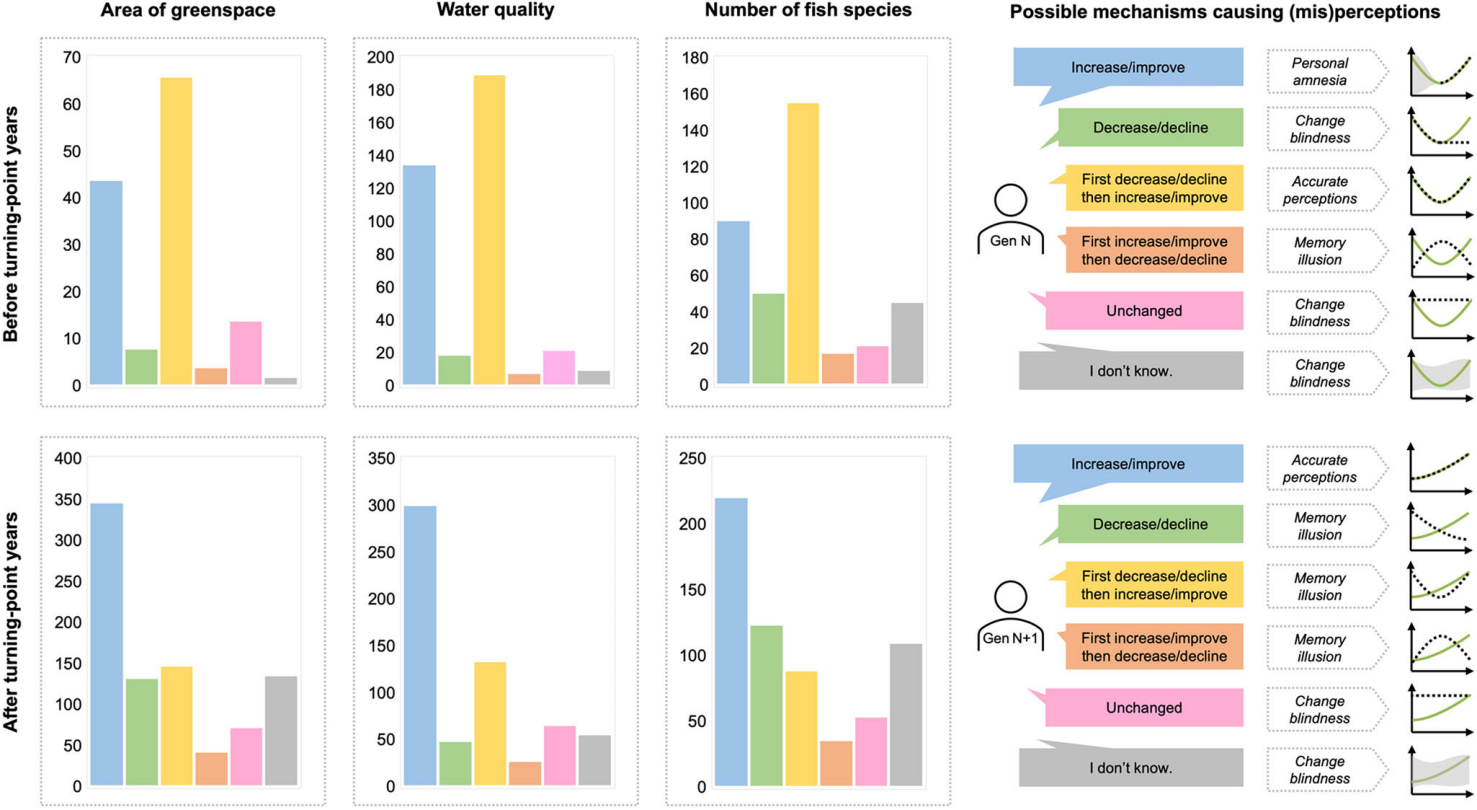
In Qunli New Town, China, perceptions (colors) of respondents (*n* = 1018) of green space area, water quality, and number of fish species relative to when respondents set their baseline ecological knowledge: (a) baseline is years before the turning point (i.e., years that mark peaks or nadirs from which a trend changes direction) (yellow, correct response) and (b) baseline is years after the turning point (blue, correct response). Graphs on the right show the different types of perceptions and misperceptions based on the conceptual model (Figure 1) (dashed line, perceptions of change; solid line, actual change; shading, uncertain perceptions).

We excluded 308 questionnaires as respondents did not provide responses to key questions concerning their perceptions of the factors driving change. Of the 1018 questionnaires left, 4 respondents responded to all perception questions but incompletely reported their sociodemographic features. They were therefore excluded from regression analyses and were used only in determinations of the different types of misperceptions (Figure 4).

The interview and questionnaire surveys were conducted with approval from the University of Oxford's Central University Research Ethics Committee (CUREC reference number R84176/RE001). All interviewees provided informed consent and were assured that interview data would remain confidential.

The accuracy of respondents’ perceptions of environmental states and trends were compared with officially reported environmental states and trends, which we took to represent the actual states and trends in the area. We evaluated the trends in 3 ecological indicators: area of greenspace, water quality, and number of fish species. We chose these because of the potentially different perceptibility of the 3 features and the availability of information on their actual changes for comparing perceptions with realities. Area of greenspace can be the most obvious feature to people who live in the area. Water quality is not as perceptible but links closely with local people's basic well‐being. Fish species are less perceivable by the majority but would be well known to people with specialist knowledge (e.g., fishers). The turning‐point years (i.e., years that mark peaks or nadirs from which a trend goes in a new direction) (Figure 2) for each of these indicators were estimated based on official documentation (Appendix S1). For greenspace, it was 2009, when the land was cleared for development and the first set of terrestrial compensatory restoration projects was completed. The turning‐point year for water quality and fish species richness was also 2009, when ecological data showed that local river restoration projects started to have a positive effect.

We asked about the previous land use in the area before land conversion took place, primarily to assess whether people recognized the previous existence of the large marsh wetland and multiple communities with farmland and fishponds. We also asked about the presence of specific plant species used in the town's compensation projects (multiple‐choice questions that included correct and incorrect options, i.e., species used and not used in local compensatory plantings).

Table 1 shows all the variables we used to evaluate the association between years of experience, quality of experience, knowledge sources, and sociodemographic characteristics and local environmental knowledge. We used 3 models to separately analyze people's overall knowledge about local environmental change and their knowledge about previous and current environmental states. Generalized linear models (GLMs) were used after data dredging was performed for variable selection (Appendix S3). The glm() function from the stats package was used to fit the GLMs, and the dredge() function from the MuMIn package was employed for model selection and comparison. Because the dependent variables were count data, we used Poisson regressions (Whitmore, 2020). Our hypothesis was that the earlier a respondent starts to experience the location, the more likely the respondent is to understand previous and current environmental states. The variable of perceived baseline year was used in all models. Factors evaluating people's experience with the previous town and the new town, their information sources, their interest in natural visits, ways to interact, and sociodemographic features were also incorporated in all 3 models.

**TABLE 1:** Variables included in the generalized linear models analyzing the effects of experience with nature and sociodemographic features on the knowledge about ecological features in Qunli, Harbin, China.

Variable	Data type^a^	Description
Dependent variables		
Overall knowledge score	Count	Out of 14 questions on environmental states before and after land conversion, the number of questions the respondent answered correctly
Knowledge score before land conversion	Count	Out of 5 questions on environmental states before land conversion, the number of questions the respondent answered correctly
Knowledge score after land conversion	Count	Out of 9 questions on environmental states after land conversion, the number of questions the respondent answered correctly
Independent variables		
Experience		
Perceived baseline year	Continuous	Year in which respondent started to experience the place
Frequency of visiting the old town	Ordinal	Frequency of visits to old Qunli town before land conversion (0, never went before development began; 1, went once; 2, went there occasionally; 3, went often; 4, lived in old Qunli Town)
Time spent out of the town in the previous year	Ordinal	Time spent out of the town in the previous year (0, never or no more than 1 month; 1, 1–3 months; 2, 3–6 months; 3, more than 6 months)
Frequency of visiting the natural area in the previous year	Ordinal	Frequency of visits to the natural area in the new town in the previous year (0, never; 1, <3 times in total; 2, <1 time per month; 3, once per fortnight; 4, once per week; 5, most days)
Time spent per visit (last year)	Ordinal	Time spent per visit during the last year (0, never been there; 1, <30 min; 2, around 30 min to 1 h; 3, over 1 h)
Interest in visiting nature	Ordinal	Interest in visiting the natural areas in general (0, very low; 1, low; 2, neutral; 3, high; 4, very high)
Interact directly with nature	Binary	Interact with nature directly by being in it, so I can touch, smell, and see it (0, no; 1, yes)
Information sources		
Relatives and friends	Binary	Acquire local environmental information from relatives or friends (0, no; 1, yes)
Printed media	Binary	Acquire local environmental information from printed media (0, no; 1, yes)
Television	Binary	Acquire local environmental information from TV (0, no; 1, yes)
Official websites or social media	Binary	Acquire local environmental information from official websites or social media (0, no; 1, yes)
Unofficial websites or social media	Binary	Acquire local environmental information from unofficial websites or social media (0, no; 1, yes)
Sociodemographics		
Age	Interval	0, 18–30 years; 1, 30–45 years; 2, 45–60 years; 3, >60 years
Education level	Ordinal	0, no education; 1, primary; 2, lower secondary; 3, upper secondary; 4, college diploma; 5, bachelor's degree; 6, master's degree; 7, doctoral degree
Local (Qunli)	Binary	0, non‐Qunli resident; 1, Qunli resident

^a^ Definitions: count variables, numeric values representing counts; continuous variables, numeric values over a range; ordinal variables, ordered categories with unequal intervals; binary variables, 2 possible values (usually 0 and 1); interval variables, numeric values with equal intervals but no true zero.

## RESULTS

### Age, experience, and sociodemographic features

Interview results (*n* = 42) showed that younger people were not always less knowledgeable about local nature and its change than older people. The amount of knowledge they reported related strongly to the number of years they had lived in or visited the locality. Respondents who lived in the area before development (2006) reported more knowledge about past environmental conditions and change than people who moved in after that year. Respondents who demonstrated more interest in nature generally tended to know more and their perceptions were more accurate, with some exceptions. Perceptions were constructed directly (e.g., through personal visits to natural areas) or indirectly (through other information sources):

I used to live near the town… and moved in after it's developed… I wouldn't choose to visit natural parks for fun, but I've been there a few times with my mom ‘cause she made me… She took many pictures of colorful flowers and shared them in our family group chat… One of my classmates at [a local university] whose family moved from the old Qunli … (s)he told me there were some grassland, bungalows, and factories (female, aged 22, student).

People's general interest in and time spent visiting local nature changed over time due to internal (e.g., acclimatization to new experiences) and external factors (e.g., a pandemic):

[My interest in local natural parks] was sky‐high when I first came here… People were curious to see what the new town looked like when the development just completed. I visited [the parks] quite often when I first came here… I observed what's been changed in the [bund] wetland… [The parks] are not as enthralling now (male, aged 59, factory worker).

[My husband and I] used to stroll in those parks years ago. Now I prefer staying indoors, [because] COVID‐19 is just so frightening (female, aged 83, retired).

Through these interviews, we found that people's information sources were heterogeneous and included direct visits or indirect information from families and friends, TV, or social media. They also showed different ways of processing the environmental information they had acquired, which led to correct and incorrect knowledge about past and current environmental conditions. Ideas about how the place had changed were sometimes generated without direct or indirect experience of it (see errors of commission in Table 3).

Results of the GLMs showed that length of experience, rather than age, predicted environmental knowledge (Table 2). Interest in visiting nature and time spent per visit significantly determined how much knowledge respondents had about local environmental situations. The effect of direct interactions with nature was marginally significant. Gaining information from (state‐run) television was associated with less accurate knowledge of the previous ecological state of the area.

**TABLE 2:** Regression‐estimated effects from 3 generalized linear models of experience with nature and sources of information about nature on environmental knowledge (*n* = 1014 survey respondents).

	Model 1 overall knowledge			Model 2 before turning point knowledge			Model 3 current conditions knowledge		
Variable	Estimate	SD	*p*	Estimate	SD	*p*	Estimate	SD	*p*
Intercept	35.450	3.329	<0.001	47.751	5.856	<0.001	31.618	4.566	<0.001
Experience									
Perceived baseline year	−0.017	0.002	<0.001	−0.024	0.003	<0.001	−0.015	0.002	<0.001
Frequency of visiting the old town				0.034	0.019	0.072	−0.044	0.015	0.004
Frequency of visiting the natural area (last year)	0.020	0.012	0.092				0.030	0.014	0.038
Time spent per visit (last year)	0.055	0.018	0.003				0.080	0.023	<0.001
Interest in visiting nature	0.061	0.016	<0.001	0.102	0.024	<0.001	0.047	0.020	0.018
Interacting directly with nature	0.065	0.035	0.067				0.077	0.044	0.083
Knowledge sources									
Relatives or friends	0.039	0.026	0.136						
TV	−0.056	0.026	0.033	−0.086	0.043	0.044			
Sociodemographics									
Age				−0.038	0.025				
Education level	0.063	0.010	<0.001	0.099	0.019	<0.001	0.043	0.012	<0.001
Local (Qunli)	−0.045	0.028	0.105	−0.072	0.044	0.104			

*Note*: Dependent variable: a composite knowledge score. Model 1 relates overall knowledge to variables related to respondents’ experience of the area, sources of knowledge, and sociodemographic characteristics. Model 2 relates to knowledge about conditions before restoration of existing natural areas and creation of new green spaces began. Model 3 relates to knowledge about current conditions. Minimum adequate models (MAMs) are presented, based on a model selection process. Blank cells are variables that are not in the MAMs.

People who lived in, or frequently visited, the old town before it was developed had significantly less knowledge about the environmental state of the new town. This finding was relevant to 3 interviewees who mentioned the loss of incentive to visit and explore the town after the removal of farmland, factories, and friends in the old town. For example,

I went there before; I worked in the pumping plant there… I don't go there now. Why would I (male, aged 88, retired engineer)?

Only education level was significantly and positively associated with people's knowledge of previous and current environmental states. Age was not significantly related to people's knowledge of environmental states. Also, respondents who lived in the new town were not significantly more or less knowledgeable about the previous state of the environment than those who did not live in the new town.

### Information processing, misperceptions, and SBS

Interviews results revealed the role of environmental information processing in engendering environmental misperceptions and SBS. Many cognitive elements had a role in information processing, including sensation, attention, learning, thinking, and memory, in SBS (Figure 2; quotations in Table 3).

**TABLE 3:** Examples of errors of omission and commission and relevant cognitive processes derived from surveys of people from Qunli New Town, Harbin, China, regarding their environmental misperceptions and cognitive processes driving misperceptions.

Error type	Cognitive process	Outcome (*n* = 1018)	Quotation example
Errors of omission	Sensation (lack of ability or opportunities to detect environmental features)	5% had no interest in visiting or learning about local natural environment 34% were unable to experience local nature much because they were newcomers	I prefer indoor activities … to outdoor ones (male, aged 23, college student). I don't know much about this place for now, but I'll know more if I live here longer (male, aged 60, construction engineer).
	Attention (e.g., change or inattentional blindness)	20% were too busy to carefully experience local nature by being around it 32% paid little attention to the natural environment during visits	I came to Qunli every day during high school … I didn't notice the change of natural surroundings. I only noticed, through our class window, that some high‐rise buildings nearby were under construction (female, aged 28, bank clerk). I traveled to cities like Beijing and Shenzhen for many times every year … In a year, I may live here for 3 months and there for 4 (female, aged 52, millionaire].
	Learning (e.g., lack of information sources to acquire new knowledge)	25% lacked knowledge sources to learn local environmental situations	I can't find any database that has recorded the previous situations in Qunli … Media also rarely reported them. Very few did but most of those pages are no longer available now (male, aged 30, junior researcher).
	Thinking (e.g., lack of knowledge to identify a species)	31% did not have sufficient ecological or scientific knowledge to identify or distinguish natural elements	We can see new types of birds flying in every couple years … I cannot identify every kind of them, but it seemed that they were in different shapes, colors (male, aged 61, former farmer). There grows a vast area of lavender in Qunli Bund. (female, aged 70, retired worker). The plant was actually sage (Salvia officinalis).
	Memory (e.g., childhood amnesia or age‐related memory loss)	16% had some environmental knowledge, but could not remember it	My dad told me he has brought me to there [the old Qunli Town] when I was a kid, but I can't remember that (male, aged 25, graduate student). There were no factories or communities … I'm not very sure. It's been years (male, aged 89, retired pump engineer).
Errors of commission	Learning (e.g., misinformation or disinformation)	37% were uncertain about their previously acquired environmental knowledge Especially, people who receive information from state‐run TV program were significantly less likely to report correct previous or current environmental situations	TV programs reported that the ecological environment in the [Songhua] river has gradually become better … In the 21st century it's never been seriously polluted [which is untrue] as the government has taken good care of it (female, aged 28, bank clerk).
	Thinking (e.g., overconfidence bias; unreality‐based speculation)	Respondents asserted they knew local environmental situations very well but reported inaccurate previous (12%) and current (16%) environmental states and had below‐average scores	This place was empty with no nature and no people living all along. That's why it was planned and developed into an urban area (male, aged 55, electrician).
	Memory (e.g., memory illusion; misattribution)	48% selected the wrong years in which restoration of the Qunli bund wetland begun	Development of the Qunli New Town started from around 2010… Around that year, the nature in the town, like the bund wetland, started to be restored” (female, aged 35, business owner).

Based on the questionnaire, all the potential options for the shape of the environmental change curve (Figure 1) were represented in the answers received (Figure 4). Overall, the majority of respondents were correct in their assessment of environmental change across all 3 environmental dimensions, despite some dimensions (e.g., area of land) being easier to observe than others (water pollution, fish species). There were more “don't know” responses for the number of fish species, which is not surprising given that this is the least observable dimension. Even so, most people (88% and 83%, respectively) felt able to give an answer. There was also a general tendency to say that things had improved across all 3 dimensions. In those who were present before the turning point, this represented personal amnesia (i.e., SBS as defined by Papworth et al. [2009]).

Although different cognitive processes led to erroneous environmental reporting, as various interviewees revealed (see quotations in Table 3), some processes appeared to help prolong respondents’ understanding of past environmental situations:

My husband often went to the previous swamp and collected wild duck eggs. I would use them to make pickled eggs … I remember those eggs were fresh and tasty because those wild ducks grew up eating fish from the Songhua River… They were much more delicious than the eggs nowadays laid by ducks raised on feed (female, aged 69, cleaner).

Some cognitive elements appeared to be particularly dominant in people's explanations of their lack of ability to describe environmental change. For example, some interviewees stated they had little to no experience with local nature due to having only recently moved to the area or lack of interest; hence, they were not able to develop a sensory engagement with the area (the first stage of perception in Figure 2). Memory was also frequently cited as an issue when interviewees failed to recall past events.

Based on interviews and questionnaire survey, we devised the framework to classify different cognitive elements underlying environmental misperceptions as errors of omission or commission shown in Table 3.

## DISCUSSION

Transformative change is required to achieve a nature‐positive future in which humanity remains safely within planetary boundaries (Díaz et al., 2019; Fischer et al., 2007; Milner‐Gulland et al., 2021). To realize such a future, environmental misperceptions must be addressed because they can lower people's expectations as to how much nature recovery is needed (Manning et al., 2006; Soga & Gaston, 2018). Using our proposed framework, environmental misperceptions are studied at the societal and cognitive levels within a local setting. For the first half of the framework (Figure 1), we used survey data to describe the extent to which local residents experienced different types of SBS (Figure 4) and identified factors influencing environmental misperceptions locally (Table 2). For the second half of the framework (Figure 2), we analyzed interview data to assess whether and how misperceptions occurred across cognitive stages and used our survey data to quantify the proportion of misperceptions attributed to each stage in this context (Table 3).

Our finding that close interaction with local nature does not necessarily translate into local knowledge of ecological states and change presents an intriguing contradiction to common assumptions about the human–nature connection (Bögeholz, 2006; Duerden & Witt, 2010). Physical engagement with green spaces is often linked to heightened environmental knowledge, but our results suggest that presence or use of such spaces may not, in itself, foster deep ecological understanding. This disconnect raises important questions about the quality, intentionality, and educational components of human–nature interactions. It may be that meaningful ecological knowledge depends not only on physical proximity but also on the individual's curiosity and interest in the natural world. Without active engagement and intention, experiences in nature may remain passive. The results thus highlight the potential value of deliberate interventions, such as interpretive signage, community workshops, or participatory conservation efforts, that encourage interest and understanding and help bridge the gap between interaction and ecological understanding.

Our study showed how important it is that more academic attention be given to the cognitive mechanisms underpinning people's experiences of nature loss and recovery. The cognitive revolution in 1950s shifted psychological study from studying human behavior to examining the many mental processes that underlie perceptions of the outside world, including sensation, attention, learning, thinking, and memory (Gardner, 1987; Miller, 2003; Núñez et al., 2019). This improved psychological understanding. We found that people's experience of nature was significantly associated with their perceptions of its state and trends. Direct and immersive personal experience can correctly shape people's environmental perceptions, whereas indirect experience with unreliable information sources can implant incorrect knowledge that deviates their perceived baselines from reality. However, experience with nature from the cognitive perspective includes many mental processes that act together to shape people's perceptions. Researchers working on nature recovery should investigate these processes.

Drawing on information processing theory (Figure 2), we found that errors can take place at diverse cognitive stages (sensation, attention, learning, thinking, and memory). Based on our proposed framework of errors of omission and commission (Table 3), we argue that it is vital to understand how misperceptions were generated at a cognitive level before designing and applying interventions to address misperceptions that are contextually meaningful and thus potentially effective. For example, strategies for people who have little motivation to be in nature need to differ from strategies for people with little ecological knowledge if the aim is to develop their understanding of nature. Further, the interventions needed for people exposed to environmental misinformation will be very distinct from those designed for people, young or old, experiencing recall failure of past environmental conditions.

Our results indicated that people's ability to mentally process changes in different natural features can vary. Respondents found it the most difficult to report change in fish species richness, even incorrectly, probably because they cannot see it. Compared with species richness, the area of greenspace may be more perceivable, but newcomers may need more time to explore the area before they can form an unbiased understanding. Water quality may be more detectable at the sensation stage in some circumstances, for example, in our context, from polluted, smelly streams to odorless ones, than in other circumstances, for example, the presence of heavy metal ions. To our knowledge, ours is the first SBS study in which perceptions of different aspects of nature were compared. There is the potential for much more research on this topic and the application of a plurality of methods, including controlled experiments.

Although our results are preliminary, it appeared that some mental processes may help preserve accurate understanding of local environmental change and thus mitigate environmental misperceptions, for example, multisensory learning and emotional memory (Dinh et al., 1999; Kensinger, 2009). This calls for extensive research input from diverse fields, for example, environmental psychology, sociology, and anthropology.

To advance understanding and mitigation of environmental misperceptions, future research should systematically investigate their various manifestations, including change blindness and memory illusion, across diverse ecological and sociocultural contexts. This involves clarifying how a misperception presents itself, examining its ecological and social consequences, identifying the cognitive and structural drivers behind it, and developing context‐sensitive strategies to address it. A place‐based approach is essential because environmental misperceptions, along with their drivers and impacts, vary according to sociocultural and geographical contexts. For example, in rapidly urbanizing regions of the Global South, a key factor contributing to SBS could be the erosion of intergenerational ecological knowledge linked to rural–urban migration (Lasisi & Ekpenyong, 2011; Rangel et al., 2024). In the Global North, digital media saturation is one potentially important driver that exacerbates memory distortions and deepens temporal disconnection from nature (Edwards & Larson, 2020). These differences underscore the importance of identifying key mechanisms, including cognitive ones, such as selective attention and psychological biases, that shape environmental misperceptions. Moreover, future work should also examine different interventions for addressing misperceptions, including immersive, multisensory environmental education, community‐based participatory initiatives, and gamified learning platforms designed to reconnect individuals with environmental realities. Interdisciplinary collaboration must underpin this research agenda. For example, sociological and anthropological perspectives can illuminate how cultural histories and collective identities shape environmental perceptions, upon which psychological experiments can be designed to test causal mechanisms and evaluate the efficacy of emotionally resonant environmental narratives in countering memory illusions. Such efforts are critical for designing evidence‐based strategies to align public understanding with environmental realities, a necessary foundation for a nature‐ and people‐positive future.

## AUTHOR CONTRIBUTIONS

The study was jointly conceptualized by all authors. Shuo Gao led the data collection. All authors contributed to data analyses, interpretation of findings, and the writing and critical revision of the manuscript.

## Supporting information



Supporting Information

Supporting Information

Supporting Information

## References

[cobi70157-bib-0001] Ackrill, R. , Igudia, E. , Olusanya, O. , & Oyalowo, B. (2023). Street level bureaucrats, policy entrepreneurship, and discretion in enforcing bans on motorcycle taxis in Lagos, Nigeria. European Policy Analysis, 9(4), 440–464.

[cobi70157-bib-0002] Agyeman, J. (2013). Introducing just sustainabilities: Policy, planning, and practice. Zed Books.

[cobi70157-bib-0003] Austin, G. , & Yu, K. (2016). Constructed wetlands and sustainable development. Taylor & Francis.

[cobi70157-bib-0004] Bögeholz, S. (2006). Nature experience and its importance for environmental knowledge, values and action: Recent German empirical contributions. Environmental Education Research, 12(1), 65–84.

[cobi70157-bib-0005] Braun, V. , & Clarke, V. (2006). Using thematic analysis in psychology. Qualitative Research in Psychology, 3(2), 77–101.

[cobi70157-bib-0006] Braun, V. , & Clarke, V. (2021). To saturate or not to saturate? Questioning data saturation as a useful concept for thematic analysis and sample‐size rationales. Qualitative Research in Sport, Exercise and Health, 13(2), 201–216.

[cobi70157-bib-0007] Brislin, R. W. (1970). Back‐translation for cross‐cultural research. Journal of Cross‐Cultural Psychology, 1(3), 185–216.

[cobi70157-bib-0008] Brislin, R. W. (1986). The wording and translation of research instruments. In W. J. Lonner & J. W. Berry (Eds.), Field methods in cross‐cultural research (pp. 137–164). Sage Publications.

[cobi70157-bib-0009] Brown, A. S. (1991). A review of the tip‐of‐the‐tongue experience. Psychological Bulletin, 109(2), 204–223. 2034750 10.1037/0033-2909.109.2.204

[cobi70157-bib-0010] Bruner, J. S. (1974). Toward a theory of instruction. Harvard University Press.

[cobi70157-bib-0011] Bryce, R. , Irvine, K. N. , Church, A. , Fish, R. , Ranger, S. , & Kenter, J. O. (2016). Subjective well‐being indicators for large‐scale assessment of cultural ecosystem services. Ecosystem Services, 21, 258–269.

[cobi70157-bib-0012] Bull, J. W. , Milner‐Gulland, E. J. , Addison, P. F. , Arlidge, W. N. , Baker, J. , Brooks, T. M. , Burgass, M. J. , Hinsley, A. , Maron, M. , Robinson, J. G. , Sekhran, N. , Sinclair, S. P. , Stuart, S. N. , zu Ermgassen, S. O. S. E. , & Watson, J. E. (2020). Net positive outcomes for nature. Nature Ecology & Evolution, 4(1), 4–7. 31686021 10.1038/s41559-019-1022-z

[cobi70157-bib-0013] Bull, J. W. , & Strange, N. (2018). The global extent of biodiversity offset implementation under no net loss policies. Nature Sustainability, 1(12), 790–798.

[cobi70157-bib-0014] Damiens, F. L. , Porter, L. , & Gordon, A. (2021). The politics of biodiversity offsetting across time and institutional scales. Nature Sustainability, 4(2), 170–179.

[cobi70157-bib-0015] Daw, T. (2010). Shifting baselines and memory illusions: What should we worry about when inferring trends from resource user interviews? Animal Conservation, 13(6), 534–535.

[cobi70157-bib-0016] Díaz, S. , Fargione, J. , Chapin, F. S., III , & Tilman, D. (2006). Biodiversity loss threatens human well‐being. PLoS Biology, 4(8), Article e277. 16895442 10.1371/journal.pbio.0040277 PMC1543691

[cobi70157-bib-0017] Díaz, S. , Settele, J. , Brondízio, E. S. , Ngo, H. T. , Agard, J. , Arneth, A. , Balvanera, P. , Brauman, K. A. , Butchart, S. H. M. , Chan, K. M. A. , Garibaldi, L. A. , Ichii, K. , Liu, J. , Subramanian, S. M. , Midgley, G. F. , Miloslavich, P. , Molnár, Z. , Obura, D. , Pfaff, A. , & Zayas, C. N. (2019). Pervasive human‐driven decline of life on Earth points to the need for transformative change. Science, 366(6471), Article eaax3100. 31831642 10.1126/science.aax3100

[cobi70157-bib-0018] Dinh, H. Q. , Walker, N. , Hodges, L. F. , Song, C. , & Kobayashi, A. (1999). Evaluating the importance of multi‐sensory input on memory and the sense of presence in virtual environments. In Proceedings IEEE Virtual Reality (Cat. No. 99CB36316) (pp. 222–228). IEEE.

[cobi70157-bib-0019] Downing, A. S. , Bhowmik, A. , Collste, D. , Cornell, S. E. , Donges, J. , Fetzer, I. , Häyhä, T. , Hinton, J. , Lade, S. , & Mooij, W. M. (2019). Matching scope, purpose and uses of planetary boundaries science. Environmental Research Letters, 14(7), Article 073005.

[cobi70157-bib-0020] Duerden, M. D. , & Witt, P. A. (2010). The impact of direct and indirect experiences on the development of environmental knowledge, attitudes, and behavior. Journal of Environmental Psychology, 30(4), 379–392.

[cobi70157-bib-0022] Edwards, R. C. , & Larson, B. M. (2020). When screens replace backyards: Strategies to connect digital‐media‐oriented young people to nature. Environmental Education Research, 26(7), 950–968.

[cobi70157-bib-0023] Engel, A. K. , Fries, P. , & Singer, W. (2001). Dynamic predictions: Oscillations and synchrony in top–down processing. Nature Reviews Neuroscience, 2(10), 704–716. 11584308 10.1038/35094565

[cobi70157-bib-0024] Essl, F. , Dullinger, S. , Rabitsch, W. , Hulme, P. E. , Pyšek, P. , Wilson, J. R. , & Richardson, D. M. (2015). Delayed biodiversity change: No time to waste. Trends in Ecology & Evolution, 30(7), 375–378. 26028440 10.1016/j.tree.2015.05.002

[cobi70157-bib-0025] Fischer, J. , Manning, A. D. , Steffen, W. , Rose, D. B. , Daniell, K. , Felton, A. , Garnett, S. , Gilna, B. , Heinsohn, R. , Lindenmayer, D. B. , Macdonald, B. , Mills, F. , Newell, B. , Reid, J. , Robin, L. , Sherren, K. , & Wade, A. (2007). Mind the sustainability gap. Trends in Ecology & Evolution, 22(12), 621–624. 17997188 10.1016/j.tree.2007.08.016

[cobi70157-bib-0026] Ford, J. D. , King, N. , Galappaththi, E. K. , Pearce, T. , McDowell, G. , & Harper, S. L. (2020). The resilience of indigenous peoples to environmental change. One Earth, 2(6), 532–543.

[cobi70157-bib-0027] Gao , S. , Bull, J. W. , Baker, J. , zu Ermgassen, S. O. , & Milner‐Gulland, E. J. (2023). Analyzing the outcomes of China's ecological compensation scheme for development‐related biodiversity loss. Conservation Science and Practice, 5(10), Article e13010.

[cobi70157-bib-0028] Gardner, H. (1987). The mind's new science: A history of the cognitive revolution. Basic Books.

[cobi70157-bib-0029] Gerson, K. , & Damaske, S. (2020). The science and art of interviewing. Oxford University Press.

[cobi70157-bib-0030] Gilbert, C. D. , & Sigman, M. (2007). Brain states: Top‐down influences in sensory processing. Neuron, 54(5), 677–696. 17553419 10.1016/j.neuron.2007.05.019

[cobi70157-bib-0031] Glaser, B. G. , & Strauss, A. L. (1967). The discovery of grounded theory: Strategies for qualitative research. Aldine.

[cobi70157-bib-0032] Hackmann, H. , Moser, S. C. , & St Clair, A. L. (2014). The social heart of global environmental change. Nature Climate Change, 4(8), 653–655.

[cobi70157-bib-0033] Hausmann, A. , Slotow, R. O. B. , Burns, J. K. , & Di Minin, E. (2016). The ecosystem service of sense of place: Benefits for human well‐being and biodiversity conservation. Environmental Conservation, 43(2), 117–127.

[cobi70157-bib-0034] Hidalgo Pizango, C. G. , Honorio Coronado, E. N. , del Águila‐Pasquel, J. , Flores Llampazo, G. , de Jong, J. , Cordova Oroche, C. J. , Reyna Huaymacari, J. M. , Carver, S. J. , del Castillo Torres, D. , Draper, F. C. , Phillips, O. L. , Roucoux, K. H. , de Bruin, S. , Peña‐Claros, M. , van der Zon, M. , Mitchell, G. , Lovett, J. , García Mendoza, G. , Gatica Saboya, L. , & Baker, T. R. (2022). Sustainable palm fruit harvesting as a pathway to conserve Amazon peatland forests. Nature Sustainability, 5(6), 479–487.

[cobi70157-bib-0035] Himberger, K. D. , Chien, H. Y. , & Honey, C. J. (2018). Principles of temporal processing across the cortical hierarchy. Neuroscience, 389, 161–174. 29729293 10.1016/j.neuroscience.2018.04.030

[cobi70157-bib-0036] Igudia, E. , Ackrill, R. , & Machokoto, M. (2022). Institutional incongruence, the everyday, and the persistence of street vending in Lagos: A demand‐side perspective. Environment and Planning A: Economy and Space, 54(6), 1256–1276.

[cobi70157-bib-0037] Jacob, C. , van Bochove, J. W. , Livingstone, S. , White, T. , Pilgrim, J. , & Bennun, L. (2020). Marine biodiversity offsets: Pragmatic approaches toward better conservation outcomes. Conservation Letters, 13(3), Article e12711.

[cobi70157-bib-0038] Jacoby, L. L. , & Whitehouse, K. (1989). An illusion of memory: False recognition influenced by unconscious perception. Journal of Experimental Psychology: General, 118(2), 126–135.

[cobi70157-bib-0039] Jarić, I. , Roll, U. , Bonaiuto, M. , Brook, B. W. , Courchamp, F. , Firth, J. A. , Gaston, K. J. , Heger, T. , Jeschke, J. M. , Ladle, R. J. , Meinard, Y. , Roberts, D. L. , Sherren, K. , Soga, M. , Soriano‐Redondo, A. , Veríssimo, D. , & Correia, R. A. (2022). Societal extinction of species. Trends in Ecology & Evolution, 37(5), 411–419. 35181167 10.1016/j.tree.2021.12.011

[cobi70157-bib-0040] Jensen, M. S. , Yao, R. , Street, W. N. , & Simons, D. J. (2011). Change blindness and inattentional blindness. Wiley Interdisciplinary Reviews: Cognitive Science, 2(5), 529–546. 26302304 10.1002/wcs.130

[cobi70157-bib-0041] Jones, L. P. , Turvey, S. T. , Massimino, D. , & Papworth, S. K. (2020). Investigating the implications of shifting baseline syndrome on conservation. People and Nature, 2(4), 1131–1144.

[cobi70157-bib-0042] Kausler, D. H. (2012). Experimental psychology, cognition, and human aging. Springer Science & Business Media.

[cobi70157-bib-0043] Kensinger, E. A. (2009). Remembering the details: Effects of emotion. Emotion Review, 1(2), 99–113. 19421427 10.1177/1754073908100432 PMC2676782

[cobi70157-bib-0044] Kerley, G. I. , Kowalczyk, R. , & Cromsigt, J. P. (2012). Conservation implications of the refugee species concept and the European bison: King of the forest or refugee in a marginal habitat? Ecography, 35(6), 519–529.

[cobi70157-bib-0045] Kerley, G. I. , te Beest, M. , Cromsigt, J. P. , Pauly, D. , & Shultz, S. (2020). The protected area paradox and refugee species: The giant panda and baselines shifted towards conserving species in marginal habitats. Conservation Science and Practice, 2(6), Article e203.

[cobi70157-bib-0046] Kuemmerle, T. , Hickler, T. , Olofsson, J. , Schurgers, G. , & Radeloff, V. C. (2012). Refugee species: Which historic baseline should inform conservation planning? Diversity and Distributions, 18(12), 1258–1261.

[cobi70157-bib-0047] Lasisi, R. , & Ekpenyong, A. S. (2011). Urbanization and loss of traditional ecological knowledge: Lessons from Rumuodomaya Community in Rivers State. International Journal of Cross‐Cultural Studies, 1(1), 54–64.

[cobi70157-bib-0109] Li, C. , Gao, S. , & Xia, L. (2021). Tourism development projects and nature loss on Xuedou Mountain, China. Oryx, 55(1), 11–11.

[cobi70157-bib-0048] Lindsay, P. H. , & Norman, D. A. (1972). Human information processing: An introduction to psychology. Academic Press.

[cobi70157-bib-0049] Lovell, S. , Johnson, A. E. , Ramdeen, R. , & McClenachan, L. (2020). Shifted baselines and the policy placebo effect in conservation. Oryx, 54(3), 383–391.

[cobi70157-bib-0050] Low, J. (2019). A pragmatic definition of the concept of theoretical saturation. Sociological Focus, 52(2), 131–139.

[cobi70157-bib-0051] Makino, H. , & Komiyama, T. (2015). Learning enhances the relative impact of top‐down processing in the visual cortex. Nature Neuroscience, 18(8), 1116–1122. 26167904 10.1038/nn.4061 PMC4523093

[cobi70157-bib-0052] Manning, A. D. , Lindenmayer, D. B. , & Fischer, J. (2006). Stretch goals and backcasting: Approaches for overcoming barriers to large‐scale ecological restoration. Restoration Ecology, 14(4), 487–492.

[cobi70157-bib-0053] Maron, M. , Quétier, F. , Sarmiento, M. , Ten Kate, K. , Evans, M. C. , Bull, J. W. , Jones, J. P. G. , zu Ermgassen, S. O. S. E. , Milner‐Gulland, E. J. , Brownlie, S. , Treweek, J. , & von Hase, A. (2024). ‘Nature positive’ must incorporate, not undermine, the mitigation hierarchy. Nature Ecology & Evolution, 8(1), 14–17. 37735564 10.1038/s41559-023-02199-2

[cobi70157-bib-0054] Maylor, E. A. (1990). Age, blocking and the tip of the tongue state. British Journal of Psychology, 81(2), 123–134. 2364243 10.1111/j.2044-8295.1990.tb02350.x

[cobi70157-bib-0055] McClenachan, L. , Matsuura, R. , Shah, P. , & Dissanayake, S. T. (2018). Shifted baselines reduce willingness to pay for conservation. Frontiers in Marine Science, 5, Article 48.

[cobi70157-bib-0056] Mesulam, M. M. (1998). From sensation to cognition. Brain: A Journal of Neurology, 121(6), 1013–1052. 9648540 10.1093/brain/121.6.1013

[cobi70157-bib-0057] Millennium Ecosystem Assessment . (2005). Ecosystems and human well‐being: Synthesis. Island Press.

[cobi70157-bib-0058] Miller, G. A. (2003). The cognitive revolution: A historical perspective. Trends in Cognitive Sciences, 7(3), 141–144. 12639696 10.1016/s1364-6613(03)00029-9

[cobi70157-bib-0059] Miller, J. R. (2005). Biodiversity conservation and the extinction of experience. Trends in Ecology & Evolution, 20(8), 430–434. 16701413 10.1016/j.tree.2005.05.013

[cobi70157-bib-0108] Milner‐Gulland, E. J. , Addison, P. , Arlidge, W. N. S. , Baker, J. , Booth, H. , Brooks, T. , Bull, J. W. , Burgass, M. J. , Ekstrom, J. , zu Ermgassen, S. O. S. E. , Fleming, L. V. , Grub, H. M. J. , von Hase, A. , Hoffmann, M. , Hutton, J. , Juffe‐Bignoli, D. , Ten Kate, K. , Kiesecker, J. , Kümpel, N. F. , & Watson, J. E. M. (2021). Four steps for the Earth: mainstreaming the post‐2020 global biodiversity framework. One Earth, 4(1), 75–87.

[cobi70157-bib-0060] Muldrow, M. , Parsons, E. , & Jonas, R. (2020). Shifting baseline syndrome among coral reef scientists. Humanities and Social Sciences Communications, 7(1), Article 42.

[cobi70157-bib-0061] Newell, A. , & Simon, H. A. (1972). Human problem solving. Englewood Cliffs.

[cobi70157-bib-0062] Núñez, R. , Allen, M. , Gao, R. , Miller Rigoli, C. , Relaford‐Doyle, J. , & Semenuks, A. (2019). What happened to cognitive science? Nature Human Behaviour, 3(8), 782–791. 10.1038/s41562-019-0626-2 31182794

[cobi70157-bib-0063] Oppenheimer, D. M. , & Kelso, E. (2015). Information processing as a paradigm for decision making. Annual Review of Psychology, 66, 277–294. 10.1146/annurev-psych-010814-015148 25559114

[cobi70157-bib-0064] O'Reilly, M. , & Parker, N. (2013). ‘Unsatisfactory saturation’: A critical exploration of the notion of saturated sample sizes in qualitative research. Qualitative Research, 13(2), 190–197.

[cobi70157-bib-0065] Ostergren, D. M. , Abrams, J. B. , & Lowe, K. A. (2008). Fire in the forest: Public perceptions of ecological restoration in north‐central Arizona. Ecological Restoration, 26(1), 51–60.

[cobi70157-bib-0066] Papworth, S. K. , Rist, J. , Coad, L. , & Milner‐Gulland, E. J. (2009). Evidence for shifting baseline syndrome in conservation. Conservation Letters, 2(2), 93–100.

[cobi70157-bib-0067] Passoni, G. , Coulson, T. , & Cagnacci, F. (2023). Celebrating wildlife population recovery through education. Trends in Ecology & Evolution, 39(2), 101–105. 38065709 10.1016/j.tree.2023.10.004

[cobi70157-bib-0068] Pauly, D. (1995). Anecdotes and the shifting baseline syndrome of fisheries. Trends in Ecology & Evolution, 10(10), Article 430. 21237093 10.1016/s0169-5347(00)89171-5

[cobi70157-bib-0069] Philippssen, J. S. , Angeoletto, F. H. S. , & Santana, R. G. (2017). Education level and income are important for good environmental awareness: A case study from south Brazil. Ecología Austral, 27(1), 39–44.

[cobi70157-bib-0070] Pisoni, D. B. (2000). Cognitive factors and cochlear implants: Some thoughts on perception, learning, and memory in speech perception. Ear and Hearing, 21(1), 70–78. 10708075 10.1097/00003446-200002000-00010 PMC3429935

[cobi70157-bib-0071] Rangel, J. M. L. , do Nascimento, A. L. B. , & Ramos, M. A. (2024). The influence of urbanization on local ecological knowledge: A systematic review. Journal of Ethnobiology and Ethnomedicine, 20(1), Article 106. 39695704 10.1186/s13002-024-00747-z PMC11657303

[cobi70157-bib-0072] Raworth, K. (2017). A Doughnut for the Anthropocene: Humanity's compass in the 21st century. The Lancet Planetary Health, 1(2), e48–e49. 29851576 10.1016/S2542-5196(17)30028-1

[cobi70157-bib-0073] Reitman, W. R. (1964). Information‐processing models in psychology. Science, 144(3623), 1192–1198. 14150317 10.1126/science.144.3623.1192

[cobi70157-bib-0074] Richardson, K. , Steffen, W. , Lucht, W. , Bendtsen, J. , Cornell, S. E. , Donges, J. F. , Drüke, M. , Fetzer, I. , Bala, G. , von Bloh, W. , Feulner, G. , Fiedler, S. , Gerten, D. , Gleeson, T. , Hofmann, M. , Huiskamp, W. , Kummu, M. , Mohan, C. , Nogués‐Bravo, D. , & Rockström, J. (2023). Earth beyond six of nine planetary boundaries. Science Advances, 9(37), Article eadh2458. 37703365 10.1126/sciadv.adh2458 PMC10499318

[cobi70157-bib-0075] Robles‐Zavala, E. , & Reynoso, A. G. C. (2018). The recreational value of coral reefs in the Mexican Pacific. Ocean & Coastal Management, 157, 1–8.

[cobi70157-bib-0076] Roediger, H. L., III. (1996). Memory illusions. Journal of Memory and Language, 35(2), 76–100.

[cobi70157-bib-0077] Roman, J. , Dunphy‐Daly, M. M. , Johnston, D. W. , & Read, A. J. (2015). Lifting baselines to address the consequences of conservation success. Trends in Ecology & Evolution, 30(6), 299–302. 26042680 10.1016/j.tree.2015.04.003

[cobi70157-bib-0078] Rui, J. R. (2023). Health information sharing via social network sites (SNSs): Integrating social support and socioemotional selectivity theory. Health Communication, 38(11), 2430–2440. 35574665 10.1080/10410236.2022.2074779

[cobi70157-bib-0079] Schacter, D. L. (1999). The seven sins of memory: Insights from psychology and cognitive neuroscience. American Psychologist, 54(3), 182–203. 10199218 10.1037//0003-066x.54.3.182

[cobi70157-bib-0080] Schacter, D. L. (2002). The seven sins of memory: How the mind forgets and remembers. Houghton Mifflin.

[cobi70157-bib-0081] Schacter, D. L. (2022). The seven sins of memory: An update. Memory, 30(1), 37–42. 33459149 10.1080/09658211.2021.1873391 PMC8285452

[cobi70157-bib-0082] Shoyama, K. , Managi, S. , & Yamagata, Y. (2013). Public preferences for biodiversity conservation and climate‐change mitigation: A choice experiment using ecosystem services indicators. Land Use Policy, 34, 282–293.

[cobi70157-bib-0083] Simon, H. A. (1978). Information‐processing theory of human problem solving. Handbook of Learning and Cognitive Processes, 5, 271–295.

[cobi70157-bib-0084] Simons, D. J. , & Levin, D. T. (1997). Change blindness. Trends in Cognitive Sciences, 1(7), 261–267. 21223921 10.1016/S1364-6613(97)01080-2

[cobi70157-bib-0086] Small, M. L. (2009). ‘How many cases do I need?’ On science and the logic of case selection in field‐based research. Ethnography, 10(1), 5–38.

[cobi70157-bib-0107] Smith, K. J. , Pierson, J. C. , Evans, M. J. , Gordon, I. J. , & Manning, A. D. (2024). Continental‐scale identification and prioritisation of potential refugee species; a case study for rodents in Australia. Ecography, 2024(9), e07035.

[cobi70157-bib-0087] Soga, M. , & Gaston, K. J. (2016). Extinction of experience: The loss of human–nature interactions. Frontiers in Ecology and the Environment, 14(2), 94–101.

[cobi70157-bib-0088] Soga, M. , & Gaston, K. J. (2018). Shifting baseline syndrome: Causes, consequences, and implications. Frontiers in Ecology and the Environment, 16(4), 222–230.

[cobi70157-bib-0089] Soga, M. , & Gaston, K. J. (2023). Global synthesis reveals heterogeneous changes in connection of humans to nature. One Earth, 6(2), 131–138.

[cobi70157-bib-0090] Soga, M. , Gaston, K. J. , Fukano, Y. , & Evans, M. J. (2023). The vicious cycle of biophobia. Trends in Ecology & Evolution, 38(6), 512–520. 36707258 10.1016/j.tree.2022.12.012

[cobi70157-bib-0091] Solso, R. L. , MacLin, M. K. , & MacLin, O. H. (2005). Cognitive psychology. Pearson Education.

[cobi70157-bib-0092] Straka, T. M. , Glahe, C. , Dietrich, U. , Bui, M. , & Kowarik, I. (2025). From nature experience to pro‐conservation action: How generational amnesia and declining nature‐relatedness shape behaviour intentions of adolescents and adults. Ambio, 54, 1165–1184. 40048120 10.1007/s13280-025-02135-7 PMC12133659

[cobi70157-bib-0093] Swanson, H. A. , Svenning, J. C. , Saxena, A. , Muscarella, R. , Franklin, J. , Garbelotto, M. , Mathews, A. S. , Saito, O. , Schnitzler, A. E. , Serra‐Diaz, J. M. , & Tsing, A. L. (2021). History as grounds for interdisciplinarity: Promoting sustainable woodlands via an integrative ecological and socio‐cultural perspective. One Earth, 4(2), 226–237.

[cobi70157-bib-0094] Teufel, C. , & Nanay, B. (2017). How to (and how not to) think about top‐down influences on visual perception. Consciousness and Cognition, 47, 17–25. 27238628 10.1016/j.concog.2016.05.008

[cobi70157-bib-0095] UN‐Habitat . (2022). Cities and nature: Planning for the future . https://unhabitat.org/sites/default/files/2022/12/white_paper_cities_and_nature_rev2.pdf

[cobi70157-bib-0096] von Stein, A. , Chiang, C. , & König, P. (2000). Top‐down processing mediated by interareal synchronization. Proceedings of the National Academy of Sciences of the United States of America, 97(26), 14748–14753. 11121074 10.1073/pnas.97.26.14748 PMC18990

[cobi70157-bib-0097] Wang, X. , Chen, H. , Chen, Z. , & Yang, Y. (2021). Women's intrasexual competition results in beautification. Social Psychological and Personality Science, 12(5), 648–657.

[cobi70157-bib-0098] Whitmore, N. (2020). R for conservation and development projects: A primer for practitioners. CRC Press.

[cobi70157-bib-0099] Wray, N. , Markovic, M. , & Manderson, L. (2007). “Researcher saturation”: The impact of data triangulation and intensive‐research practices on the researcher and qualitative research process. Qualitative Health Research, 17(10), 1392–1402. 18000078 10.1177/1049732307308308

[cobi70157-bib-0100] Yin, R. K. (2009). Case study research: Design and methods (5th ed.). Sage Publications.

[cobi70157-bib-0101] Yuille, A. , & Kersten, D. (2006). Vision as Bayesian inference: Analysis by synthesis? Trends in Cognitive Sciences, 10(7), 301–308. 16784882 10.1016/j.tics.2006.05.002

[cobi70157-bib-0102] Zeyer, A. , & Kelsey, E. (2013). Environmental education in a cultural context. In R. B. Stevenson , M. Brody , J. Dillon , & A. E. Walls (Eds.), International handbook of research on environmental education (pp. 206–212). Routledge.

[cobi70157-bib-0103] Zheng, W. , Wan, A. K. Y. , Chen, Z. , Clark, A. , Court, C. , Gu, Y. , Park, T. , Reynolds, J. , Zhang, X. , Li, L. , & Lee, T. M. (2024). Use of consumer insights to inform behavior change interventions aimed at illegal pet turtle trade in China. Conservation Biology, 38(5), Article e14352. 39248772 10.1111/cobi.14352

[cobi70157-bib-0104] Zhu, X. , Zhang, Y. , & Zhao, W. (2020). Differences in environmental information acquisition from urban green—A case study of Qunli National Wetland Park in Harbin, China. Sustainability, 12(19), Article 8128.

[cobi70157-bib-0105] zu Ermgassen, S. O. , Howard, M. , Bennun, L. , Addison, P. F. , Bull, J. W. , Loveridge, R. , Pollard, E. , & Starkey, M. (2022). Are corporate biodiversity commitments consistent with delivering ‘nature‐positive’ outcomes? A review of ‘nature‐positive’ definitions, company progress and challenges. Journal of Cleaner Production, 379(Pt. 2), Article 134798.

