# Supplementary material for: Addressing environmental misperceptions for nature recovery
Source: Conserv Biol. 2025 Oct 18;40(2):e70157. doi: 10.1111/cobi.70157 (PMC13036313; doi:10.1111/cobi.70157)
Supplement: Supplementary file 1 — Supporting Information [file COBI-40-e70157-s001.pdf]

APPENDICES

Official Evidence of Local Environmental Trends (in Chinese)

sina 新闻中心 新浪首页 > 新闻中心 > 国内新闻 > 正文

污染水位低使松花江鱼类锐减 十网九空江开无鱼

http://www.sina.com.cn 2005年04月17日10:26 东北新闻网

到了吃开江鱼的时候，可连日来，一些打鱼者纷纷反映，松花江里难觅鱼踪，有的时候甚至一整天都打不到一斤鱼。据哈尔滨市农委渔政处负责人介绍，松花江水连年被污染及今年水位偏低，是导致鱼量减少的主要原因。

12日9时许，记者在哈尔滨市松花江北岸"马家船口"附近看到，只有二三艘长约10米的小渔船在江边缓慢地划行，其他20几艘小渔船都停靠在江岸上。正在划行的每艘打渔船上都站着两个人，其中一个人划船，一个人拿着鱼网忙着捕鱼。可是，每次他们收网时几乎都没有收获。其中，一位40余岁的男子上岸后告诉记者，前几天，来这里捕鱼的人还很多，但由于江里的鱼少，几乎捕不到鱼，所以许多捕者这几天都没有下江。

Photo not available

消费热点 中国网络歌曲排行榜 打造校园生活第一黄页 公司治理EMBA 宝石公园 时尚新生活

家住哈尔滨市松浦镇的老赵今年44岁，多年来靠种地为生，每年开江的时候，他都到松花江打鱼来贴补家用。据老赵讲，10年前他在这里每天都可以打到10多斤鱼，而且鱼的种类很多。可这两年来，他明显感觉到江里的鱼越来越少，捕鱼越来越难。常常是"十网九空"。今年开江以来，他已经连续打七八天鱼了，最多一天，他也就打了不到2斤"嘎牙子"。

据哈尔滨市农委渔政处相关负责人薛军介绍，据记载，松花江里最早共有79种鱼。但在1990年，哈尔滨市环境监测中心站和哈尔滨市水产资源管理站的科研人员在松花江却里只采集到56种鱼。而研究人员在2000年开江期到当年8月中旬的采集研究中发现，松花江里鱼的种类又下降到了34种，到目前种类可能更少。基本可以确定，目前在松花江里，甲鱼、日本七鳃鳗、大白鱼，以及鳊花已经绝迹了，鳌花、狗鱼、青鱼、雅罗、乌苏里白鲑等保护鱼类也基本绝迹。薛军说，导致鱼量锐减的主要原因是，松花江哈尔滨段每天要承载上游和哈尔滨的污水580吨，其中近百吨是未经处理的污水，而且连续十几年松花江开江时水位偏低。

目前，哈尔滨市渔政部门正采用限量发放捕鱼证、组织人工增殖放流鱼苗、减少江水污染等方法，希望近早恢复松花江的鱼量。(新华网)[编辑： 龙秋秀]（来源：东北新闻网）

2011年06月06日 星期一 收藏本站

www.cyol.com

往期回顾 新闻列表 返回目录 ＜上一期 下一期＞ 中青报系

换一种方式“吃水”

哈尔滨：重新“发现”松花江

本报记者 吕博雄 实习生 王茜 通讯员 李兵 《中国青年报》（2011年06月06日 04 版）

哈尔滨市道外区民主乡村民王月梅一直守着松花江打渔，“一年到头也赚不到啥钱。”她根本没想到，随着松花江湿地的开发，自己家门口最近也变成了风景区。现在，一起打渔的邻居都在忙着适应新职业——有的改行当了导游；有的筹备渔具，开展垂钓旅游服务。据说，同乡胜利村小七队的59户村民，抓住湿地旅游开发的机会，干脆整村搬进由“小别墅”群组成的现代都市村庄，干起了“农家乐”。

这种改变，也发生在哈尔滨市松花江沿岸的千家万户当中。哈尔滨市下辖的137个乡镇中，濒临松花江的41个，占全市乡镇总数的30%。随着近几年当地湿地旅游建设的推进，沿岸农民也正在实现自身角色的转变。

南方许多大城市江河被渠化的今天，松花江沿哈尔滨绵延120公里，依然保持着蜿蜒曲折的原生状态，实在是一笔天赐的资源。“横向对比，哈尔滨近年来的经济总量已落后于兄弟城市，唯有找准方向，才能迎头赶上。”黑龙江省委常委、哈尔滨市委书记盖如垠在接受中国青年报记者独家专访时说，要培育建设周期较长的新经济增长点，更要利用城市自身优势，发展旅游是绝佳选择——以旅游来惠民生，以旅游来调结构，以旅游来促进环境保护，“我们决定，不拘泥于GDP指数，也不搞‘形象工程’、‘政绩工程’，而是实实在在地让老百姓过上看得见、摸得着、用得上的好日子。”

最新调研证明，时至今日，在哈尔滨，松花江流域内形成的湿地面积达468平方公里，仅自然形成湿地就占全市土地面积的5.4%，是哈尔滨恢复松花江水生态与城乡一体化、老工业基地经济结构调整之间的最佳契合点。靠山吃山，靠水吃水，百年哈尔滨如今决定换一种方式“吃水”，使这个城市与松花江一同迈入人水和谐的时代。

源于发现的“水经济”

上个世纪八九十年代，歌曲《太阳岛上》风靡全国，曾勾起人们对哈尔滨太阳岛风景区的无限想往。可大多数人不知道，太阳岛风景区只是哈尔滨市已开发及正在开发的11处类似湿地之一。而风景独特的金河湾湿地、阳明滩湿地等景区资源，一直不为人知。3年前，很多湿地景区甚至人迹罕至。去年，全年湿地游的游客也仅有56万人。目前，哈尔滨对水资源的利用率在20%左右，远低于欧洲滨水城市60%的标准。

这是遗憾，也是机会。研究了这些问题之后，去年8月，履新不久的哈尔滨市委书记盖如垠在探察松江湿地的船上，即兴抒怀七律一首，以“辽阔松江何许言，滨城得景靓其间”的诗句，表达了对哈尔滨湿地资源新发现的惊喜。他提出，努力打造中国最大原生态多样性城市湿地景观，使“湿地旅游”成为哈尔滨市继“冰雪节”之后的第二张城市名片。

这发现，也得到外界的认可。前不久，哈尔滨荣获了由亚太旅游联合会颁发的“大美湿地城市”称号。

“水”字与“冰”字，笔画只差两点儿。但作为一个城市的发展思路来讲，两种定位，市民完全是两重感受。以往，人们熟悉“冰城”哈尔滨，浪漫、激情，旅游主要局限于以冰雪节为主打产品的冬季，只有几个月的活跃期，相关就业、经济发展同时受到局限；现在，哈尔滨要打造“冰城夏都”，把水的四季样态，逐一呈现在全国游客面前。已经开通的15条湿地旅游线路，将使哈尔滨旅游季延长至八九个月。

哈尔滨市旅游局局长杨杰说，从全国看，很少有其他城市，特别是北方城市，具有如此独特、鲜明的内涵型水生态资源——大大小小滩岛20余个，总面积超过245平方公里。大小滩岛形成了全国最大的城中原生态多样化湿地。

《松花江哈尔滨城区段百里生态长廊总体规划》编制人员黄利群、许士国等曾对哈市的湿地生态环境进行过详尽的调查统计。结果显示，城市湿地主要植被群系有十余种植物，其中国家级保护植物就有3种。湿地已查明的鱼类有37种，占黑龙江全省鱼类总数的35%。

如今这个城市正以“旅游即城市，城市即旅游”的气魄和紧迫感，在丰富自己的新发现。投资2.54亿元的五项沿江湿地路网工程年内就将建成，到时候游客驱车从主城区抵达湿地景区，将从1小时缩短为20分钟；总投资100余亿元的松花江避暑城也已开建。在全市宾馆接待能力不足的现状下，这个城市已开始动员大小招待所提高规模、层次，甚至新办家庭旅馆可以暂时免税经营。

“一江居中、两岸繁荣”

提及儿时的松花江，曾在环保部门工作十余年的原哈尔滨市环保局副局长李贵友不胜唏嘘。上世纪50年代，江水清澈、水产丰富，他和伙伴常常到江边游泳、垂钓。随着上世纪50年代及改革开放后沿江工业的建设，松花江变了。

“在上世纪70年代，松花江主体水质能够达到三类水；80年代末期，只能达到四类水了；到90年代中期，基本上就不能游泳了。”李贵友说。哈市农委渔政处提供的数据显示，1988年的渔业产量，只有1959年的五分之一。也就是说，松花江鱼类资源量，30年间减少了80%。

这些，刺痛了2009年刚刚上任的哈尔滨市委书记盖如垠。市委连续召开了七、八、九次全会，用科学发展的思路和目标统一思想。

哈尔滨拥有松花江、呼兰河等众多大小河流、湖泊，水资源丰富。由于历史原因，江北一直是洪涝灾害多发区，水资源开发利用程度低，而且松花江干流和支流污染、滩涂无序开发、河道占用等问题日益严重。

如何做好“水”文章？2009年，盖如垠首次提出了“以水定城”理念，即城市发展以水系网络为骨架，以松花江沿岸产业带开发、呼兰河以及肇兰新河综合治理为重点，道路、水电等基础设施随水系湖泊建设、整治构建，形成“北国水城”新格局——计划利用松花江天然丰富的水资源，向北引水，通过水生态环境修复和综合整治、污染控制与水系连通，建设人工水系湖泊、沿岸绿化、河湖连通的河网体系，并建设8座铁路桥、78座公路桥和68座步行桥，完成水城的主干骨骼建设。

松花江北岸堤防和堤顶路工程将同步启动。总投资超过70亿元，涉及河道111公里，集防洪、交通、景观、岸线整治、生态修复于一体，堤防防洪能力全部达到百年一遇标准，成为哈尔滨"水城"战略的重要安全屏障。

经过几年的建设，松花江水质重回三类水标准，江边又有了垂钓者。从2009年起，哈尔滨完成了松花江流域污染防治规划24个项目，总投资近28亿元，松花江哈尔滨段的排污主力"三沟一河"得到有效治理。仅去年一年，松花江干流出境水质主要污染指标就累计降低12.9％。

松花江的水质经历了从清澈到污染，又逐渐好转的过程，松花江湿地也经历了由退化到修复的过程。而这个转变过程，也被认为是哈尔滨发展思路的转变。哈尔滨市长林铎在前不久的一次环境会议上就强调，环境保护要与转方式、调结构，以及惠民生、促和谐结合起来。

生态的改善，使哈尔滨增添了开发利用松花江及其湿地的信心。在哈尔滨"一江居中、两岸繁荣"的发展战略中，松花江第一次被赋予重要的意义。

具体产业规划"一横一纵一园"中的"一纵"，就是以松花江为依托的旅游产业带。松花江及流域湿地的开发寄托了哈尔滨太多的期待，以湿地旅游为主打的旅游业被定位为战略性支柱产业之一，被视为经济结构调整的新增长点。

"因地制宜发展旅游业，上符合国家经济结构调整战略，下可提高居民收入。"盖如垠说。按照哈尔滨的旅游发展规划，未来3～5年，哈尔滨计划建设松花江湿地十景、松花江十景、太阳岛十景，形成湿地景观链以及包括旅游、休闲度假、房地产等在内的沿江产业带。

开发与不开发之间

"冰城夏都"的定位，掀起了当地人对松花江开发建设的热情。但怎么建设，市委、市政府一直在开发与不开发，甚至多种开发方案中权衡。

湿地因其对环境保护的特殊意义，在生态学上通常被誉为"地球之肾"。哈尔滨的干部们都知道，对湿地旅游资源的开发，保护是前提——只在邻近城市的滩岛进行小部分的开发，且核心区域是不能进入的。最大限度保护湿地原生态才能实现可持续发展。

在松花江湿地开发进程中，哈尔滨坊间曾流传这样的段子：在长岭湖湿地及月亮湖湿地的规划中，有关部门曾费尽周折引进了多家开发商共同开发，方案报给市领导时，却被全盘推翻。得到的答复是，"开发不好，不如先养鱼"。

黑龙江省环保厅总工程师赵宴滨介绍，湿地在抵御洪水、调节径流、蓄洪防旱、净化水源等方面都有其它系统不可替代的作用。但由于区域气候的变化、水资源过度开发、工农业生产活动的持续干扰等因素，在哈尔滨人对湿地的概念和功用不甚明了的年代，松花江湿地曾一度呈现出中度甚至重度退化。就在去年，还有企业向松花江成吨排放工业废水。就在今年5月初，松江江流域的一片200亩的湿地还被毁湿开荒。

如今开发湿地是否意味着更大程度破坏？如何在开发的同时，保护湿地、"收复"湿地？

在开发与不开发之间，哈尔滨有自己的权衡。

狗岛是松花江泛洪区自然形成的梭形岛，目前正在开发建设，而其生态恢复工程总投资就达2.4亿元，包含水系开挖、护岸、进出口水闸、环岛路等。开发旅游项目计划投资却只有1.13亿元，主要建设湿地高尔夫球场、马术俱乐部、驿站广场等。

在这个城市，去年曾产生一年之内4次改版哈尔滨地图的"城建速度"，在"奋起追赶，努力晋位"的新一届市委班子的口号下，哈尔滨以整个城市的名义提出"城市即旅游，旅游即城市"的理念以加快建设。但这个城市并没有急躁和忽视自然规律。盖如垠说，相比于多数国内城市河流渠化、硬化，原生态是松江湿地的最大特色，不能拿出最佳的规划方案，不如搁置开发，着力改善生态。

为此，急性子的哈尔滨人干出了一些慢性子的事。虽然松江湿地旅游开发非常紧迫，哈尔滨市水务局对于被破坏湿地的治理方针仍以自然恢复为主。湿地修复是一个系统工程，加之哈尔滨周围的湿地由于前期破坏比较严重，植物群落经过演变、生息，到产生最适合湿地生长的物种，一般来说，需要10余年的时间才能逐渐恢复。

粗犷的哈尔滨人也学会了在湿地开发前精心地约法三章。最近出台的《松花江湿地旅游管理办法》，开宗明义就强调了湿地保护优先的原则。此外，还有诸如滨江湿地风景区"六不准"等管理框架，沿岸农民也被要求必须以村集体为单位成立旅游公司，杜绝小规模的零散开发。

开发与不开发，都体现了城市科学发展的要义。不久前，第二届"冰城夏都号——哈尔滨市民宣传哈尔滨旅游大篷车"已经驶向全国，哈尔滨人藉此希望，让全世界的游客共同来感受人水和谐的新哈尔滨。

本报哈尔滨6月5日电

中国新闻网 首页 → 新闻中心 → 国内新闻 字号： 大 中 小

松花江休养生息再现生机 久违珍稀鱼种重新出现

2012年04月19日 13:52 来源：中国环境报 参与互动(0)

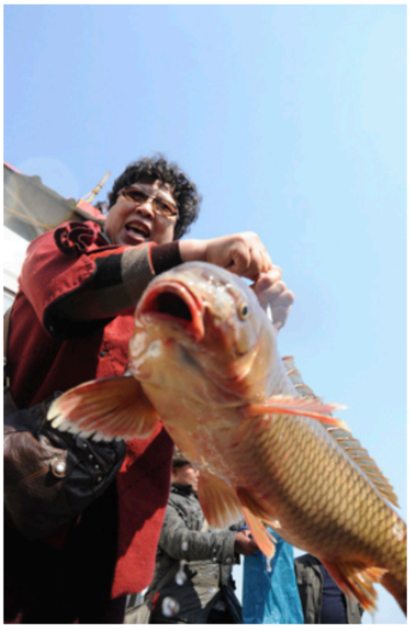

在哈尔滨九站码头，一位渔民兴奋地向记者展示刚刚捕到的大江鲤，竟然有18斤4两重。 中国环境报记者 吴殿峰 摄

开江鱼来了。

这几天在刚刚开江的松花江哈尔滨江段，渔民们正忙着捕捞开江鱼。

“今年开江可是遇上了开门红，一网下去，能捕到近百斤的开江鱼。”一位渔民兴奋地告诉记者。

在哈尔滨松花江滨洲铁路桥段的江面，渔民王顺开根本没想到，在渔网中来回跳跃的，竟是鲫花鱼和鳌花。

这些一度在松花江内消失的珍稀鱼类再次出现，令王顺开兴奋不已。

又见开江鱼

“现在松花江水质好，可以放心吃”

记者来到松花江哈尔滨九站码头，这里人头攒动，岸边的一个个大水槽子里分别装着鲶鱼、鲫鱼、鲤鱼等。围观的市民说，好多年没见到这么大的江鲤了。

卖鱼的渔民告诉记者，这都是今早刚打上来的开江鱼，其中一尾江鲤重达18斤4两，这是他近几年来捕到的最大的开江鱼。

他说:“这是松花江土生土长的开江鱼，现在松花江水质好，可以放心吃。”

松花江是黑龙江人民的母亲河，早在一千年前，春季开江就已经成为民俗中的一件盛事。每年这个时候，在松花江边生活的居民都盼着能品尝到鲜美的开江鱼。

住在松花江江边的老一辈都知道，那时候，在江边“小九站”经常有不少穿着“水衩”的打鱼人。沿着江边有好多渔村，江里有乌苏里白鲑、鲟鱼、鳌花、黑斑狗鱼……但从上世纪60年代中后期开始，沿松花江中上游建设的大型重化工业相继投产，沿江的城镇快速膨胀，污染日益严重，松花江里的江鲤、江鲫以及鲫花、鳊花、胡罗等市民常说的“三花五罗十八子”，有的难觅踪影，有的濒临绝迹。

而如今，随着松花江流域治污力度的不断加大，一些久违的鱼种又能见到。

生机重现松花江

鱼类大量出现，是松花江水环境变好的最有力佐证

在哈尔滨防洪纪念塔广场的开江祈福现场秀上，渔民和赫哲族人正在用松花江开江水洗手，两口百印大锅摆在红砖搭起的炉灶上，锅内雪白的鱼肉冒着腾腾热气，一派赫哲族渔家生活场景。

热闹的场景下，还有许多市民和志愿者在清理母亲河畔的垃圾，他们将杂物一一收进垃圾袋内。人们在欢庆的时刻，并没有忘记更好地保护母亲河。

以“生机重现”4个字来概括今天的松花江再恰当不过。

黑龙江省环境监测中心站对松花江流域内55个河流断面监测的结果显示，2011年流域内水质达标率比2008年提高了21.8个百分点，松花江干流的溶解氧上升显著，鱼类种类和产量相应增加。

哈尔滨市环境监测中心站水质监测曲线图清楚地记录着近几年松花江水质改善的过程:2006年、2007年、2008年，松花江哈尔滨段Ⅲ类水质的达标监测断面仅为20%；从2009年开始，Ⅲ类水质的达标断面开始增至60%，2010年达到80%；2011年，这一数据提升至100%，松花江哈尔滨段5个监测断面水质均为Ⅲ类。

中国水产科学研究院黑龙江水产研究所研究员姜作发介绍说，这些年，通过污染治理，松花江珍稀鱼类已经开始重现，且其种群数量还在不断增加。

2010年，姜作发在进行松花江干流调查时，采集的鱼类种类已经达到64种。

鱼类大量出现，是松花江水环境变好的最有力佐证。

休养生息带来水清鱼跃

“江水越来越清澈，我的心也越来越敞亮”

在江畔住了几十年的许大爷每年开江时都会守在江边:“这些年，看到城市迅速发展，日子越过越好，江水越来越清澈，我的心也越来越敞亮。”

“十一五”以来，沿松花江流域的吉林省、黑龙江省全面实施松花江流域水污染防治规划，给予松花江充分的人文关怀，不断减轻其污染负荷，恢复生态系统的活力，使松花江再现盎然生机。

除了中央财政投入外，黑龙江省级财政近几年累计投入奖补资金14.4亿元，大力支持松花江流域水污染防治项目建设，流域环境得到了有效改善。

环境保护部部长周生贤多次视察黑龙江省松花江流域水污染防治工作，提出要使松花江成为全国江河湖泊休养生息的样板和探索环保新道路的示范区，促进全流域的可持续发展与和谐稳定。黑龙江省省长王宪魁提出，“十二五”期间要继续推进松花江流域水污染治理，全面改善支流水环境质量，不断提高干流断面水质达标率。

“十二五”期间，黑龙江省计划总投资约150亿元，重点解决面源污染问题。到“十三五”结束时，松花江全流域的环境质量将基本恢复到三四十年前水平。(记者吴殿峰)

水质改善鱼类增多江风清新 记忆里的松花江又回来了

新浪黑龙江
09月01日 14:36

关注

摘要： 这几年，松花江哈尔滨段能吸引那么多人前来游玩，主要与水质的改善有关。记者从环保、渔政等部门了解到，通过对松花江流域风险企业进行搬迁和关闭，管控进入松花江的污染源，并进行投放鱼苗、延长禁渔期等生态修复，松花江的水质越来越好，鱼类种类数量也得到恢复，没有了腥臭，江风清新，游人如织。

原标题：水质改善鱼类增多江风清新

记忆里的松花江又回来了

来源：生活报

生活报讯（记者张立王萌）本报8月31日刊发"冰城最美沿江全景图步步倾心"的报道后，引起了广泛关注。记者走访发现，这几年，松花江哈尔滨段能吸引那么多人前来游玩，主要与水质的改善有关。记者从环保、渔政等部门了解到，通过对松花江流域风险企业进行搬迁和关闭，管控进入松花江的污染源，并进行投放鱼苗、延长禁渔期等生态修复，松花江的水质越来越好，鱼类种类数量也得到恢复，没有了腥臭，江风清新，游人如织。

20年游泳爱好者

讲述松花江水质变化

8月31日，宽阔的松花江从哈尔滨市区流过，岸边是成排的垂钓者，台阶上坐着很多人，看着荡漾的江水、吹着江风，非常惬意。

"10年前，我有一次出差来过哈尔滨，在当地朋友的带领下去了防洪纪念塔，那时松花江没有现在宽，江水有股腥臭味，不好闻。"来自贵州的高先生告诉记者，他现在退休了，又带着老伴来哈尔滨避暑，这次来到江边，江风卷来一阵江水的味道，像刚割过的草地，鲜甜鲜甜的。

除了游客，在松花江游了20多年泳的市民米长珍，对松花江的水质更有感触。今年67岁的米长珍，2016年曾获得第十六届哈尔滨国际冬泳邀请赛女子老年组冠军，她告诉记者，这几年松花江的水

质好多了，游泳后简单冲洗一下就行。"前些年冬天，江上有清沟，不结冰，都是各河沟流进的污水。夏天也能在江面上看到一条污水带顺江而下，'泾渭分明'。"米长珍说，她们游泳虽然避开污水带，但是水质也不行，有一股味儿，难闻，尤其是靠近江南的水，她们都去江北一带游泳。"这几年在哪游泳都行了，水质都很清澈，随便畅游。"今年，她们几个游泳爱好者已经多次横渡

松花江，还从十四道街码头游到了港务局码头。

多举措修复松花江鱼类增多

踏访中，市民张大爷介绍："我和几个老伙伴儿每天都要来江边垂钓，只

要往江边一坐，手杆一支，什么烦恼都没了。现在还有渔政部门修护治理，不仅水质环境得到大幅度提升，小鱼小虾等品种也更加丰富，儿时记忆里的松花江又回来了。”

记者从省农业农村厅渔政管理局获悉，据初步统计，今年已经增殖放流各种鱼类苗种8646万尾，其中包括施氏鲟、达氏鳇、哲罗鲑、细鳞鲑、大麻哈鱼等。从2019年起，松花江禁渔期为5月16日至7月31日，首次延长30天。与此同时，2020年初以来，我省组织开展“渔政亮剑2020”系列专项执法行动，环保志愿者也加入其中，收缴违禁网具12.6万延长米，收缴地笼等违禁渔具5918（张顶、套）。

通过这一系列行动，松花江生态逐渐恢复。黑龙江水产研究所调查发现，近年来，哲罗鱼、苏里白鲑、黑斑狗鱼、七鳃鳗等7种珍稀冷水鱼在松花江不同江段频频出现，对水生态环境要求苛刻的冷水鱼重新回归松花江，表明水质已得到明显改善，水生态环境得到初步恢复。来自省环境监测中心站的鱼类分析显示，松花江流域鱼体污染残留较少，鱼类生存状态较好，食用相对安全。

松花江干流哈尔滨江段水质全优

近些年，有关部门对沿线城市松花江流域风险企业进行了搬迁和关闭，目前沿江的化工、制药、焦化等企业大多已经撤出。记者从省环保厅获悉，我省深入实施水污染防治行动计划，目前已成功消除阿什河、倭肯河、梧桐河劣V类水体。2019年的环保公报显示，松花江水系水质与2018年相比，I~III类水质比例升高6.4个百分点，劣五类比例降低1.3个百分点。

今年一季度，哈市13个国考断面优良（I~III类）比例69.23%，超过国家考核要求7.7个百分点，国考断面全面消除劣V类。据了解，2008年松花江哈尔滨段三类水质的达标监测断面仅为20%，2011年这一数据提升至100%并一直持续，2019年1至9月份，松花江干流水质持续为优。

更多区域喝上干净的松花江水

据专家介绍，松花江干流水质达到三类，即可作为集中式生活饮用水地表水源地二级保护区。2013年，哈市水务局组织哈工大市政环境工程学院和哈供水集团多次检测结果显示，哈尔滨松花江水源地原水水质达到国家三类地表水水体条件，109项指标均在正常范围之内，符合饮用水供水条件。

根据哈市水源规划，哈市2014年11月份启动了松花江水源工程建设。根据当时的计划，哈南、哈西、松北、呼兰区等区域将饮用松花江水。2018年，12万松北居民已喝上优质松花江水。今年7月，松花江水源供水工程（净水管线）开始施工，竣工后万宝大道、利民一带的居民也有望告别地下水，喝上松花江水。目前，哈市已将松花江水源地取水口上移项目列入计划。上移后，取水口远离城区，水质将会有所提高。

Perceptions of Environmental Change Survey

<u>Introduction & Consent</u>

- Hello, my name is [redacted]. I am an independent doctoral researcher at the University of Oxford in the UK. Thank you for taking the time to answer this questionnaire.
- My research seeks to explore your understanding of environmental changes in Qunli, including those related to the Songhua River.
- We are surveying people who are residents over the age of 18 and currently living in Qunli New Town, Harbin, China.

How this questionnaire will work

- This survey will take around 15-20 minutes to complete.
- If you choose to participate, all your responses will be kept confidential and anonymous. We will not collect any information that can be used to identify you. No third parties, including governmental or non-governmental organisations, will have access to the information you share with us.
- You do not have to answer any question you are uncomfortable with, and you can choose to withdraw at any time.
- The collected information will be stored in a secure database, accessible only by password. The information collected is for academic use only. I will analyse the information, and the results will be presented as part of my doctoral thesis. Some results may also be published internationally in academic papers, at conferences, and on online blogs.
- In accordance with the University of Oxford's procedures for ethical approval of research involving human participants, this study has been reviewed and has received ethical clearance through a subcommittee of the University's Central University Research Ethics Committee (Ethics Approval Reference: R84176/RE001).

If you have any questions

- If you have any concerns or questions about the research, please get in touch with me, and I will do my best to assist.
- If you remain dissatisfied or wish to make a formal complaint, I can provide you with the contact details of the Research Ethics Committee at the University of Oxford.

0. Are you happy to take part in this survey?

○ Yes
○ No

<u>Section A: Environmental Changes in Qunli & the Songhua River</u>

The first part of this questionnaire asks about environmental changes in the natural areas of Qunli New Town, including the Songhua River, in Harbin, China. Please answer based on what you already know—no need to search for information, as that could affect the validity of the results. This survey is anonymous, so it's perfectly fine if you don't know the answers to some questions. Just give your best answer if you're unsure.

1. What was Qunli New Town mostly converted from? You can tick more than one answer.

○ I don't know
○ A cleared flat plain
○ Some communities, bungalows, and factories
○ A big marsh with farmland and fishponds
○ A natural woodland with a protected national wetland park
○ A large mall with extensive parking areas
○ Other, please specify: ___________

2. Around which year did the Qunli Bund Wetland restoration project begin?

○ I don’t know
○ 2007
○ 2010
○ 2013
○ 2016
○ 2019

3. In which year did you first become aware of the local situation in the Qunli area (either because you moved here or were old enough to notice changes in your surroundings)? ___________

4. Starting from the year you first became aware of the local situation and thinking up until now, how has the area of natural habitats (figure below) within the Qunli area changed?

○ Increasing
○ Decreasing
○ First decreasing, then increasing
○ First increasing, then decreasing
○ Unchanged
○ Other, please specify: ___________
○ I don’t know

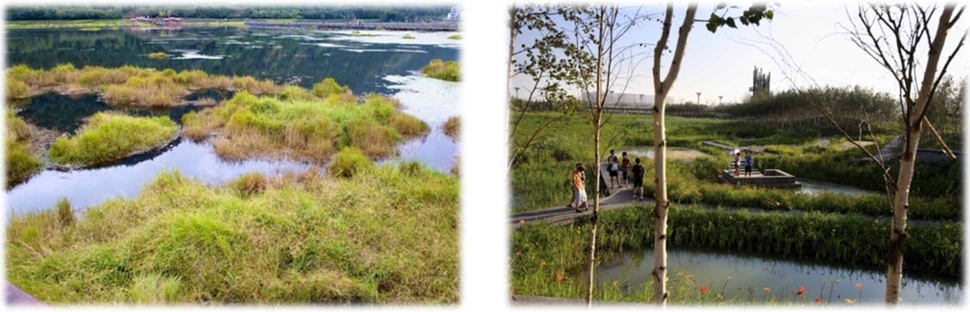

A natural habitat provides space for plants or animals to live in. It can be naturally occurring (left) or artificially created following proper procedures (right).

5. In which year did you first start to get to know about the situation in Songhua River (Harbin Section) (either because you moved to places nearby, or you were old enough to notice changes in your surroundings)? ___________

6. Compared to the year you first became aware of the local situation, how has the water quality of the Songhua River changed up to the present?

○ Improving
○ Declining
○ First declining, then improving
○ First improving, then declining
○ Unchanged
○ Other, please specify: ___________
○ I don’t know

7. Compared to the year you first became aware of the local situation, how has the number of fish kinds (figure below) in the Songhua River changed up to the present?

○ Increasing
○ Decreasing
○ First decreasing, then increasing
○ First increasing, then decreasing
○ Unchanged
○ Other, please specify: ___________
○ I don’t know

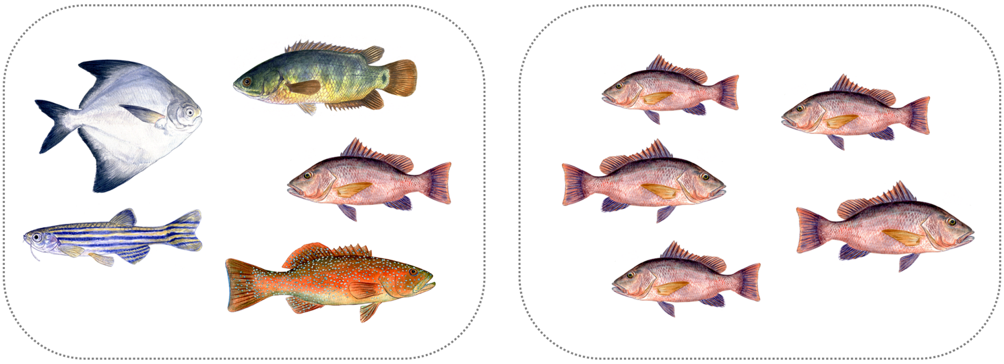

The number of fish kinds is different from the number of fish. Both panels above have 5 fish, while there are 5 kinds of bird in the left but only 1 kind in the right.

8. Which of the plants listed below were planted during the development of Qunli New Town?

○ Yes ○ No ○ I don’t know

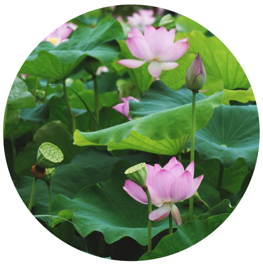

Lotus

○ Yes ○ No ○ I don’t know

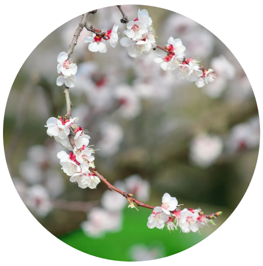

Siberian apricot

○ Yes ○ No ○ I don’t know

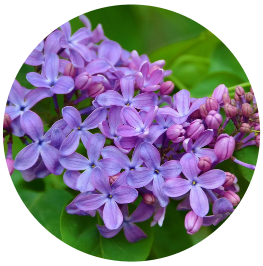

Lilac

○ Yes ○ No ○ I don’t know

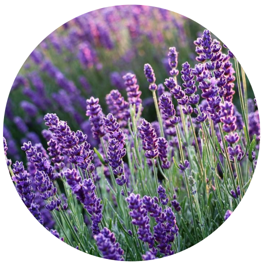

Lavender

○ Yes ○ No ○ I don’t know

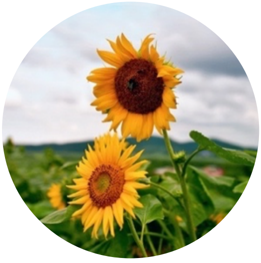

Sunflower

○ Yes ○ No ○ I don’t know

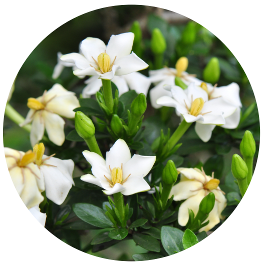

Gardenia

○ Yes ○ No ○ I don’t know

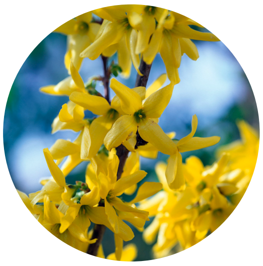

Forsythia

○ Yes ○ No ○ I don’t know

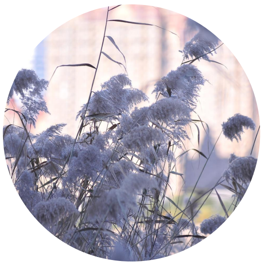

Reed flower

○ Yes ○ No ○ I don’t know

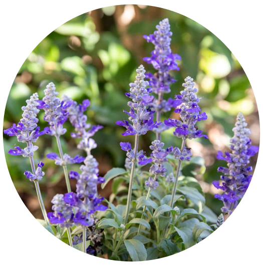

Sage

9. How confident are you in your answers to these questions?

○ Very confident
○ Somewhat confident
○ Not very confident
○ Not confident at all

10. Please explain the reasons for your answers to this question.

○ I know a lot about this place.

○ I don’t know much about this place.

11. If you don’t know much about this place, please indicate the reasons (you can select more than one).

○ I have no interest to visit or learn knowledge about local natural environment.
○ I am unable to know local nature a lot because I am new to the town.
○ I am too busy to fully appreciate the local nature, even though I am around it.
○ I have paid little attention to the natural environment during my visits.
○ I lack sources of knowledge to learn about the local environmental situation.
○ I do not have sufficient ecological or scientific knowledge to identify or distinguish natural elements.
○ I used to have some environmental knowledge, but I can’t remember it now.
○I feel uncertain about the environmental knowledge I previously acquired from media, other people, or various information sources.

12. How do you know about the environmental conditions in Qunli? You can select more than one option.

○ My own experience
○ Relatives or friends
○ Print media (e.g., newspapers)
○ Television
○ Official pages (government websites or official social media accounts)
○ Non-official pages (personal websites or unofficial social media accounts)
○ I don’t have any knowledge of the local environmental conditions in Qunli
○ Other, please specify: ___________

13. How often have you visited the natural areas in Qunli over the last 12 months?

○ Never
○ Less than three times in total
○ Less than once a month
○ About once a fortnight
○ About once a week
○ Most days

14. For each visit, how much time do you usually spend in the natural areas (including the Songhua River) in Qunli?

○ Never been there
○ Less than 30 minutes
○ Around 30 minutes to an hour
○ Over an hour

15. How interested are you in visiting the natural areas in Qunli?

○ Very low
○ Low
○ Neutral
○ High
○ Very high

16. How do you interact with the natural environment and features in Qunli (including the Songhua River)? You can select more than one option.

○ I look at them from a distance (e.g., through the windows of my flat, car, or bus).
○ I interact with nature directly by being within it, so I can touch, smell, and see it.
○ I don’t really interact with nature at all in this area.

17. Before the development of Qunli New Town began (around 2006), how often did you visit the old Qunli area?

○ I have never been to the old Qunli area before the development began.
○ I visited onone or a few times in total before the development began.
○ I visited from time to time before the development began.
○ I visited very often before the development began.
○ I lived in the old Qunli Area before the development began.
○ I can't remember.

Section B: Socio-demographic information

In this section, I would like to gather some information about you. These questions will help us understand how different backgrounds might influence perceptions of environmental changes in Qunli. Your responses will remain confidential and will only be used for research purposes.

18. What is your gender?
○ Male
○ Female
○ Other
○ Prefer not to say

19. How old are you?
○ 18-30
○ 31-45
○ 46-60
○ Over 60
○ Prefer not to say

20. What is the highest level of education you have completed?
○ No education
○ Primary
○ Lower secondary
○ Upper secondary
○ College diploma
○ Bachelor's degree
○ Master's degree
○ Doctoral degree
○ Prefer not to say

21. What is your current sector of work?
○ Department, organisation, or enterprise head
○ Technician or professional
○ Clerk
○ Business or social service worker
○ Agriculturalist
○ Manufacturing worker
○ Other, please specify: ____________
○ Prefer not to say

22. Did you have to change your job because of the development of Qunli New Town?
○ Yes
○ No
○ Not sure
○ Prefer not to say

23. If yes or not sure, what was your previous sector of work before the development of Qunli New Town?

○ Department, organisation, or enterprise head
○ Technician or professional
○ Clerk
○ Business or social service worker
○ Agriculturalist
○ Manufacturing worker
○ Other, please specify: ____________
○ Prefer not to say

24. During the last year, what was your approximate average monthly income?

○ Less than ¥1,000
○ ¥1,000 - ¥5,000
○ ¥5,000 - ¥10,000
○ ¥10,000 - ¥20,000
○ More than ¥20,000
○ Prefer not to say

25. During the last year, how long in total did you spend outside Qunli New Town, whether for work or personal reasons?

○ Never or no more than 1 month
○ 1-3 months
○ 3-6 months
○ Over 6 months

26. Which of the following religions do you identify with?

○ I don't have a religion
○ Buddhism
○ Taoism
○ Christianity
○ Islam
○ Other, please specify: ____________
○ Prefer not to say

27. What is your ethnicity?

○ Han
○ Manchu
○ Korean
○ Hui
○ Other, please specify: ____________
○ Prefer not to say

<u>Thank You</u>

- Thank you for taking the time to participate in this survey. Your input will provide valuable insights into how different residents perceive local environmental changes.
- If you have any concerns about any aspect of this study, please don't hesitate to contact me at [redacted]. You can expect an acknowledgment of your concern within 7 working days.
- If you remain dissatisfied or wish to make a formal complaint, please contact the Chair of the Medical Sciences Interdivisional Research Ethics Committee at the University of Oxford. You can reach them via email at ethics@medsci.ox.ac.uk.
