# Supplementary material for: Addressing environmental misperceptions for nature recovery
Source: Conserv Biol. 2025 Oct 18;40(2):e70157. doi: 10.1111/cobi.70157 (PMC13036313; doi:10.1111/cobi.70157)
Supplement: Supplementary file 2 — Supporting Information [file COBI-40-e70157-s002.pdf]

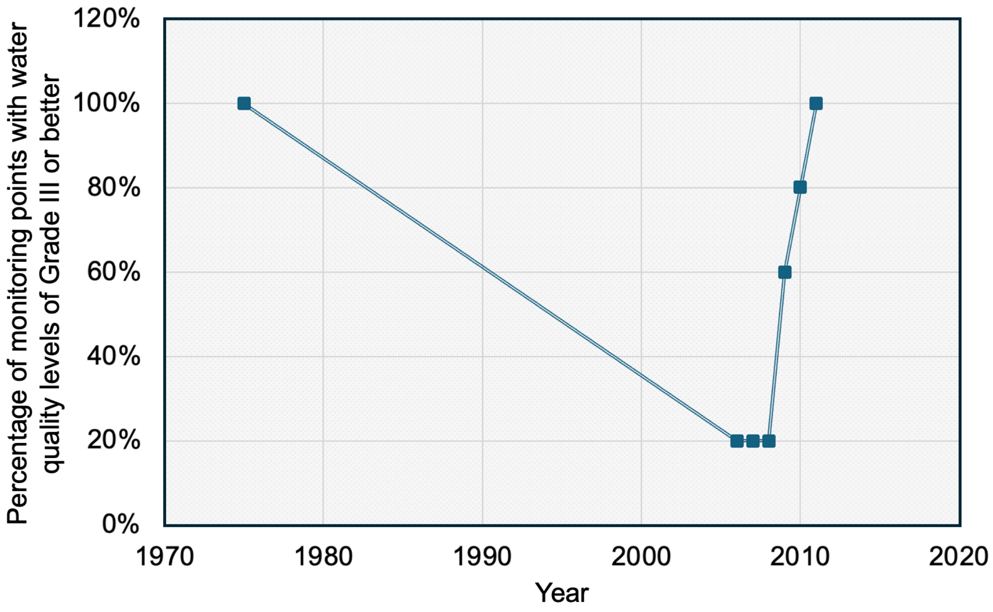

Fig. 1: The change in water quality in Songhua River (Harbin Section). Data points were collected from local official reports (see Appendix S1). Grade-III water is defined as lightly polluted water that can be used for farmland irrigation, industrial cooling and other purposes that do not come into direct human contact. The midpoint of 1975 is used to represent the reported state during the 1970s for clarity in the figure.
