# Supplementary material for: Addressing environmental misperceptions for nature recovery
Source: Conserv Biol. 2025 Oct 18;40(2):e70157. doi: 10.1111/cobi.70157 (PMC13036313; doi:10.1111/cobi.70157)
Supplement: Supplementary file 3 — Supporting Information [file COBI-40-e70157-s003.pdf]

Table C.1: Model comparison results for determining Minimum Adequate Models (MEMs). AICc: Corrected Akaike Information Criterion; RVI: Relative Variable Importance; Delta: Delta AICc.

	(Int)	Age	Baseline	Direct	Edu	Freq	Freq_pre	Friends	Interest	Official	Out	Printed	Local	Time	TV	Unofficial	AICc	Delta
(1) Overall knowledge																		
14559	35.4502	NA	-0.0170	0.0645	0.0626	0.0200	NA	0.0386	0.0614	NA	NA	NA	-0.0447	0.0547	-0.0557	NA	4483.2140	0.0000
14495	35.4977	NA	-0.0170	0.0641	0.0661	0.0209	NA	NA	0.0621	NA	NA	NA	-0.0473	0.0566	-0.0523	NA	4483.3930	0.1796
12495	35.4692	NA	-0.0170	0.0696	0.0642	NA	NA	0.0425	0.0656	NA	NA	NA	NA	0.0608	-0.0585	NA	4483.4450	0.2316
12511	34.6345	NA	-0.0166	0.0645	0.0649	0.0148	NA	0.0412	0.0605	NA	NA	NA	NA	0.0570	-0.0598	NA	4483.8140	0.5999
14543	36.2741	NA	-0.0174	0.0709	0.0624	NA	NA	0.0409	0.0675	NA	NA	NA	-0.0323	0.0601	-0.0553	NA	4483.9970	0.7835
14591	36.9168	NA	-0.0177	0.0647	0.0638	0.0212	-0.0125	0.0389	0.0620	NA	NA	NA	-0.0449	0.0553	-0.0563	NA	4484.1110	0.8967
12431	35.5076	NA	-0.0170	0.0693	0.0683	NA	NA	NA	0.0667	NA	NA	NA	NA	0.0633	-0.0549	NA	4484.1270	0.9134
12447	34.6274	NA	-0.0166	0.0640	0.0689	0.0155	NA	NA	0.0613	NA	NA	NA	NA	0.0591	-0.0563	NA	4484.3220	1.1084
14527	36.9447	NA	-0.0177	0.0643	0.0674	0.0221	-0.0123	NA	0.0627	NA	NA	NA	-0.0475	0.0572	-0.0528	NA	4484.3240	1.1101
14496	36.0213	-0.0156	-0.0172	0.0619	0.0594	0.0215	NA	NA	0.0625	NA	NA	NA	-0.0435	0.0569	-0.0504	NA	4484.3320	1.1177
14815	35.1091	NA	-0.0168	0.0674	0.0648	0.0210	NA	0.0411	0.0624	-0.0262	NA	NA	-0.0446	0.0544	-0.0531	NA	4484.3790	1.1647
14479	36.3706	NA	-0.0174	0.0707	0.0661	NA	NA	NA	0.0687	NA	NA	NA	-0.0344	0.0624	-0.0516	NA	4484.4680	1.2540
14528	38.1231	-0.0208	-0.0183	0.0614	0.0588	0.0232	-0.0164	NA	0.0634	NA	NA	NA	-0.0426	0.0578	-0.0504	NA	4484.5220	1.3085
14555	35.7814	NA	-0.0171	NA	0.0626	0.0223	NA	0.0382	0.0653	NA	NA	NA	-0.0446	0.0639	-0.0542	NA	4484.5480	1.3342
14560	35.8664	-0.0122	-0.0172	0.0628	0.0577	0.0205	NA	0.0350	0.0617	NA	NA	NA	-0.0420	0.0551	-0.0539	NA	4484.6060	1.3917
12527	36.7993	NA	-0.0176	0.0701	0.0653	NA	-0.0108	0.0429	0.0664	NA	NA	NA	NA	0.0616	-0.0589	NA	4484.6150	1.4013
Null	1.8780	NA	NA	NA	NA	NA	NA	NA	NA	NA	NA	NA	NA	NA	NA	NA	4766.8000	283.5860
Full	37.3559	-0.0190	-0.0179	0.0650	0.0588	0.0231	-0.0142	0.0361	0.0636	-0.0234	-0.0077	0.0020	-0.0415	0.0549	-0.0529	0.0043	4491.4000	8.1860
RVI		0.43	1	0.68	1	0.6	0.39	0.52	1	0.33	0.3	0.27	0.53	0.99	0.74	0.27		
(2) Pre-turning point knowledge																		
10412	47.7506	-0.0380	-0.0236	NA	0.0988	NA	0.0338	NA	0.1021	NA	NA	NA	-0.0719	NA	-0.0864	NA	3453.7340	0.0000
10475	45.3698	NA	-0.0224	NA	0.1080	NA	0.0403	0.0627	0.0982	NA	NA	NA	-0.0781	NA	-0.0954	NA	3453.8230	0.0895
14508	47.0414	-0.0391	-0.0232	NA	0.0964	NA	0.0324	NA	0.0917	NA	NA	NA	-0.0700	0.0366	-0.0815	NA	3453.9740	0.2407
10411	45.5599	NA	-0.0225	NA	0.1140	NA	0.0407	NA	0.1006	NA	NA	NA	-0.0815	NA	-0.0901	NA	3454.0100	0.2766
10476	47.2985	-0.0329	-0.0234	NA	0.0958	NA	0.0344	0.0538	0.0998	NA	NA	NA	-0.0702	NA	-0.0915	NA	3454.1790	0.4454
8364	46.2865	-0.0439	-0.0229	NA	0.1007	NA	0.0322	NA	0.0984	NA	NA	NA	NA	NA	-0.0933	NA	3454.3630	0.6294
14507	44.8019	NA	-0.0222	NA	0.1122	NA	0.0396	NA	0.0904	NA	NA	NA	-0.0800	0.0353	-0.0855	NA	3454.3740	0.6399

Table C.1 (*continued*)

	(Int)	Age	Baseline	Direct	Edu	Freq	Freq_pre	Friends	Interest	Official	Out	Printed	Local	Time	TV	Unofficial	AICc	Delta

(2) Pre-turning point knowledge																		
12460	45.5879	-0.0449	-0.0225	NA	0.0982	NA	0.0308	NA	0.0877	NA	NA	NA	NA	0.0381	-0.0878	NA	3454.4590	0.7248
14571	44.6989	NA	-0.0221	NA	0.1068	NA	0.0393	0.0586	0.0890	NA	NA	NA	-0.0769	0.0322	-0.0909	NA	3454.4850	0.7510
10416	47.1597	-0.0370	-0.0233	0.0577	0.0989	NA	0.0335	NA	0.0953	NA	NA	NA	-0.0732	NA	-0.0868	NA	3454.6420	0.9078
10479	44.8246	NA	-0.0222	0.0597	0.1078	NA	0.0398	0.0620	0.0912	NA	NA	NA	-0.0792	NA	-0.0957	NA	3454.6530	0.9196
8428	45.8813	-0.0384	-0.0227	NA	0.0975	NA	0.0328	0.0559	0.0961	NA	NA	NA	NA	NA	-0.0985	NA	3454.6810	0.9476
14572	46.6883	-0.0344	-0.0231	NA	0.0938	NA	0.0331	0.0492	0.0903	NA	NA	NA	-0.0686	0.0339	-0.0865	NA	3454.6970	0.9630
10415	44.9889	NA	-0.0223	0.0610	0.1137	NA	0.0402	NA	0.0934	NA	NA	NA	-0.0826	NA	-0.0904	NA	3454.7800	1.0467
14476	51.2562	-0.0493	-0.0253	NA	0.0950	NA	NA	NA	0.0945	NA	NA	NA	-0.0660	0.0393	-0.0813	NA	3454.8580	1.1246
10380	52.2013	-0.0486	-0.0258	NA	0.0975	NA	NA	NA	0.1058	NA	NA	NA	-0.0678	NA	-0.0867	NA	3454.8960	1.1620
Null	0.8936	NA	NA	NA	NA	NA	NA	NA	NA	NA	NA	NA	NA	NA	NA	NA	3629.0000	175.2660
Full	46.2845	-0.0326	-0.0229	0.0383	0.0957	0.0025	0.0329	0.0487	0.0871	-0.0009	0.0088	-0.0076	-0.0705	0.0289	-0.0808	-0.0281	3465.4000	11.6660
RVI		0.6	1	0.35	1	0.28	0.68	0.45	1	0.27	0.3	0.28	0.61	0.46	0.75	0.3		
(3) Current conditions knowledge																		
4287	31.6176	NA	-0.0153	0.0772	0.0433	0.0300	-0.0441	NA	0.0475	NA	NA	NA	NA	0.0796	NA	NA	3871.2110	0.0000
4799	31.1144	NA	-0.0150	0.0784	0.0448	0.0297	-0.0426	NA	0.0479	NA	-0.0160	NA	NA	0.0782	NA	NA	3871.9620	0.7509
12479	31.1792	NA	-0.0150	0.0785	0.0441	0.0307	-0.0444	NA	0.0484	NA	NA	NA	NA	0.0771	-0.0358	NA	3872.0440	0.8330
4283	31.9956	NA	-0.0154	NA	0.0434	0.0328	-0.0439	NA	0.0523	NA	NA	NA	NA	0.0902	NA	NA	3872.2250	1.0144
4543	30.8658	NA	-0.0149	0.0808	0.0464	0.0313	-0.0419	NA	0.0489	-0.0344	NA	NA	NA	0.0789	NA	NA	3872.2990	1.0883
6335	32.1385	NA	-0.0155	0.0773	0.0415	0.0337	-0.0442	NA	0.0481	NA	NA	NA	-0.0313	0.0777	NA	NA	3872.4210	1.2103
4351	31.6592	NA	-0.0153	0.0773	0.0409	0.0296	-0.0443	0.0257	0.0469	NA	NA	NA	NA	0.0784	NA	NA	3872.6220	1.4115
4288	32.4992	-0.0149	-0.0157	0.0754	0.0369	0.0314	-0.0471	NA	0.0481	NA	NA	NA	NA	0.0796	NA	NA	3872.6410	1.4300
12991	30.6404	NA	-0.0148	0.0798	0.0456	0.0303	-0.0429	NA	0.0489	NA	-0.0167	NA	NA	0.0757	-0.0373	NA	3872.6980	1.4871
4795	31.5191	NA	-0.0152	NA	0.0448	0.0324	-0.0425	NA	0.0528	NA	-0.0155	NA	NA	0.0890	NA	NA	3873.0650	1.8547
6847	31.6444	NA	-0.0153	0.0785	0.0429	0.0336	-0.0427	NA	0.0486	NA	-0.0167	NA	-0.0331	0.0762	NA	NA	3873.0760	1.8651
4800	32.1277	-0.0180	-0.0155	0.0763	0.0372	0.0312	-0.0462	NA	0.0487	NA	-0.0178	NA	NA	0.0781	NA	NA	3873.1260	1.9158
20671	31.7164	NA	-0.0153	0.0774	0.0425	0.0302	-0.0444	NA	0.0471	NA	NA	NA	NA	0.0792	NA	0.0127	3873.1410	1.9304
5055	30.4183	NA	-0.0147	0.0818	0.0476	0.0309	-0.0406	NA	0.0493	-0.0328	-0.0155	NA	NA	0.0777	NA	NA	3873.1430	1.9326
12475	31.5781	NA	-0.0152	NA	0.0441	0.0335	-0.0442	NA	0.0533	NA	NA	NA	NA	0.0880	-0.0342	NA	3873.1550	1.9447
5311	31.4898	NA	-0.0152	0.0777	0.0432	0.0302	-0.0441	NA	0.0477	NA	NA	-0.0074	NA	0.0795	NA	NA	3873.2030	1.9920
Null	1.4102	NA	NA	NA	NA	NA	NA	NA	NA	NA	NA	NA	NA	NA	NA	NA	4006.1000	134.8890
Full	31.3327	-0.0121	-0.0151	0.0821	0.0381	0.0355	-0.0440	0.0286	0.0500	-0.0357	-0.0180	0.0074	-0.0257	0.0714	-0.0372	0.0237	3881.4000	10.1890
RVI		0.34	1	0.67	0.96	0.82	0.95	0.34	0.91	0.35	0.42	0.27	0.33	0.99	0.38	0.29		
